# Mechanisms of Differential Resource Uptake and Translocation in *Agaricus bisporus*

**DOI:** 10.1111/1462-2920.70222

**Published:** 2026-01-08

**Authors:** Mădălina M. Vîta, Timo L. G. van Veghel, Lubos Polerecky, Nicole N. van der Wel, Desmond D. Eefting, Jack B. M. Middelburg, Robert‐Jan Bleichrodt

**Affiliations:** ^1^ Department of Earth Sciences Utrecht University Utrecht the Netherlands; ^2^ Microbiology, Department of Biology Utrecht University Utrecht the Netherlands; ^3^ Department of Medical Biology, Electron Microscopy Centre Amsterdam, Amsterdam UMC Location University of Amsterdam Amsterdam the Netherlands; ^4^ GeoLab Utrecht University Utrecht the Netherlands

**Keywords:** *Agaricus bisporus*, fructification, mushrooms, mycelium, mycelium network architecture, nutrients and water translocation, stable isotope tracing

## Abstract

Mushroom‐forming fungi form interconnected networks of hyphae and cords that cooperate to colonise their environment and feed developing mushrooms. To fully enable this cooperation, transport across the network is essential for the collection and delivery of resources when and where needed. It is not known to what extent, and with which resources, specific parts of the network contribute to feeding the developing mycelium and mushrooms. In this study, we investigated the translocation patterns of water, carbon and nitrogen in *Agaricus bisporus*, using stable isotope tracers. Ammonium‐derived ^15^N, but not glucose‐derived ^13^C, was consistently transported from the centre to the periphery of colonies in axenic culturing conditions. External wicking along the hyphae is likely a major translocation mechanism. Throughout the compost bed, there was ^13^C and ^15^N transport towards the mycelium colonising the casing and the developing mushrooms. Moreover, the top compost layer contributed most to feeding the growing mushrooms, irrespective of the mycelium network architecture. Within cords, we found five cell types of which one is likely specialised for nutrient transport. Overall, these findings provide new insights into nutrient uptake and translocation mechanisms in *A. bisporus*. This knowledge may ultimately enable enhanced feeding of mushrooms to improve production efficiency.

## Graphical abstract

Tracer translocation happens to growing mycelium and mushrooms, but less so within the substrate. Mushrooms take tracers predominantly from the top layer of compost. Cords facilitate nutrient transport via specialized vessel hyphae.

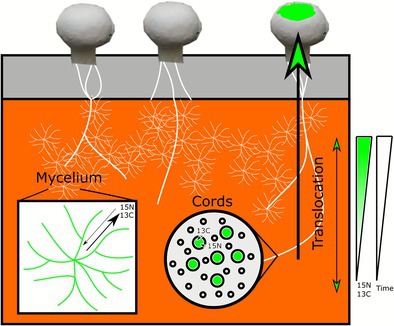

## 1 Introduction

Edible mushrooms, that is, the fruiting bodies of basidiomycete fungi, have been part of our diet since ancient times as both a source of nutrition and medicine (Wasser 2010; Bertelsen 2013). Nowadays, the cultivation of edible mushrooms offers many environmental advantages over other crops. Firstly, most mushrooms are produced on organic waste streams, such as agricultural residues, manure and wood/sawdust (Girotto and Piazza 2022; Kuyper et al. 2021), thereby reducing the environmental impact of these materials. Additionally, mushroom cultivation has a lower environmental footprint compared to other forms of food production. For instance, mushrooms are grown vertically in stacked parallel beds, which allows for a more efficient use of space (Dhar 2017), and can be grown on non‐arable land. Moreover, their cultivation requires less water than plant crops. For instance, 15.2 L of water per kilogram of mushrooms is required compared to more than 800 L to produce a kilogram of green‐house cultivated fruits and 250 L for a kilogram of vegetables (SureHarvest, n.d.; Hoekstra 2012). Furthermore, mushroom cultivation is considered as a relatively environmentally friendly food production method due to its relatively low emission of greenhouse gases (Grimm and Wösten 2018; Leiva et al. 2015). Although, at least white button mushroom production requires unsustainably sourced peat‐based casing soil, governments are phasing out the use of peat. Currently, alternative casing soil formulations are being investigated and tested in industry.

One of the most important edible mushrooms produced worldwide is *Agaricus bisporus*, the white button mushroom, or champignon (Royse et al. 2017; Atila et al. 2021). Commercially produced button mushrooms undergo a cultivation process involving two distinct composting phases, each characterised by specific microbial activities and temperature changes (Jurak et al. 2014). In phase I, the mixture of manure, wheat straw, gypsum and water undergoes fermentation, during which the compost reaches high temperatures of up to 80°C (Gerrits 1988), allowing thermophilic microorganisms to break down complex molecules (e.g., (hemi)cellulose). This is followed by phase II, where the compost cools down and is conditioned by microbial removal of ammonia (Gerrits 1988). During this phase, fungi and bacteria continue to degrade complex molecules and inhibit pathogens (Straatsma et al. 1989, 1994). Following this, in phase III, *A. bisporus* spawn is inoculated, and during the spawn running time, xylan, cellulose and lignin are partially degraded (Jurak 2015; Jurak et al. 2015). In phase IV, a peat casing layer is added and allowed to be colonised. In order to induce the formation and growth of fruiting bodies, temperature and CO_2_ levels are lowered through cooling and ventilation (Jurak 2015). A key limitation in achieving more than two full and healthy flushes of mushrooms in *A. bisporus* production is the inefficient resource utilisation by the fungus (Jurak 2015). However, little is known about the specific mechanisms of nutrient uptake, distribution and translocation within the mycelial network and compost bed, or the factors influencing nutrient utilisation by the mushrooms.

The growth of *A. bisporus* is marked by the formation of an interconnected mycelium through apical extension, subapical branching, and hyphal anastomosis (Glass et al. 2004; Read et al. 2010; Fleißner and Herzog 2016; Harris 2008), ultimately resulting in the development of aggregates of hyphae, often referred to as fungal cords (Cairney 1990). These structures enable long‐distance resource (re)distribution across the network (Herman et al. 2020; Tlalka, Fricker, and Watkinson 2008; Tlalka, Bebber, et al. 2008) primarily carbon, nitrogen, phosphorus and water (Fukasawa et al. 2024; Cairney 1992; Boddy 1999; Bebber et al. 2007) likely due to larger vessel hyphae within these cords (Herman et al. 2020). For example, the amino acid analogue amino isobutyric acid (AIB) has been specifically observed moving from compost to the growing colony margin and to developing mushrooms (Herman et al. 2020). This nutrient transport strategy is particularly advantageous in heterogeneous environments, where resources are unevenly distributed. For instance, when the colony margin encounters a new resource‐rich area, nutrients are transported from the colony centre to this new location, facilitating fast colonisation and efficient resource utilisation (Heaton et al. 2010).

While local growth can be sustained through direct nutrient uptake or intrahyphal diffusion of nutrients (Tegelaar and Wösten 2017), long‐distance transport of nutrients through the network is required for sustained growth at the colony periphery and mushroom formation (Herman et al. 2020). This is especially relevant when nutrients are locally not available or not available in sufficient amounts (e.g., during growth on nutrient poor soil or during mushroom formation (Sonnenberg et al. 2022)). The mechanism behind this transport is hyphal tip‐directed mass flow of cytoplasm (Eamus and Jennings 1984; Lew 2005). Pressure differences along the hyphae can be generated through differential osmolyte uptake (Lew 2005), internal osmolyte synthesis, external osmotic potentials (Muralidhar et al. 2016), or growth (Heaton et al. 2010; Lew 2011, 2019). In theory, water uptake at the centre of the colony would build up and maintain the necessary pressure gradient, while water release at the periphery is required to enable flow (Luard 1982). Mathematical modelling supports a mechanism of hyphal extension to enable sufficient water exit from the translocation pathway to allow mass flow of cytoplasm (Heaton et al. 2010). The flow is driven by an internal pressure gradient, in the direction from a high‐pressure to a low‐pressure area within the network. Indeed, filamentous fungi exhibit higher pressures in the colony centre than in the periphery (Luard and Griffin 1981; Thompson et al. 1985; Eamus and Jennings 1986). Out of the above four mechanisms that generate pressure differences, growth is the most important mechanism during standard conditions (Heaton et al. 2010).

In the present study, we used stable isotope tracers (^13^C and ^15^N) to assess which parts of the *A. bisporus* mycelial network take up and transport nitrogen and carbon and where these are transported to. In order to test whether the mechanisms underlying transport involve hyphal tip‐directed mass flow of cytoplasm, we sought to link nutrient and water movement within the mycelium. To this end, different types of cultures were used, ranging from axenic to compost‐based mesocosms. Deuterated water was used in an attempt to trace the water translocation patterns, while ^15^N‐ammonium chloride was used to trace nitrogen and carbon was traced either with ^13^C‐labelled glucose or fluorescently labelled 2‐deoxy‐D‐glucose (Bleichrodt and Wösten 2022). We used simple isotopic tracers in order to focus on the uptake and transport capabilities by the fungus rather than substrate degradation. In axenic cultures there was a consistent ammonium‐derived nitrogen translocation through living mycelium. However, the same trend was not detected for the glucose‐derived carbon. We hypothesize that, besides growth, wicking also drives nutrient transport across the fungal network, where wicking is defined as ‘the spontaneous flow of a liquid, driven by capillary forces’ (Kissa 1996). In compost‐based cultures, we found that tracers derived from compost primarily accumulated in the casing while the mycelium was colonising it. Mushrooms mainly derived tracers from the top compost layer, regardless of the density of cords within the compost. Additionally, using SEM‐nanoSIMS imaging, we identified five cell types within cords: (i) small and (ii) large cells devoid of cytoplasm, (iii) large cells with small internal membrane structures in which tracer‐derived carbon and nitrogen accumulated, (iv) small cells with homogeneous distribution of labelled carbon (C) and nitrogen (N), and (v) cells with organelles containing inclusion bodies, rich in phosphorous (P) and oxygen (O) or a combination of C, N and sulphur (S), and rarely a combination of all five, while the cytosol contained a homogeneous distribution of P, N, C and S. These specific cell types may play distinct roles in resource storage and transport. Overall, our findings contribute to a deeper understanding of nutrient uptake and translocation mechanisms in *A. bisporus*, providing a foundation for future efforts to optimise mushroom production strategies.

## 2 Experimental Procedures

### 2.1 Experimental Setups

**FIGURE 1:**
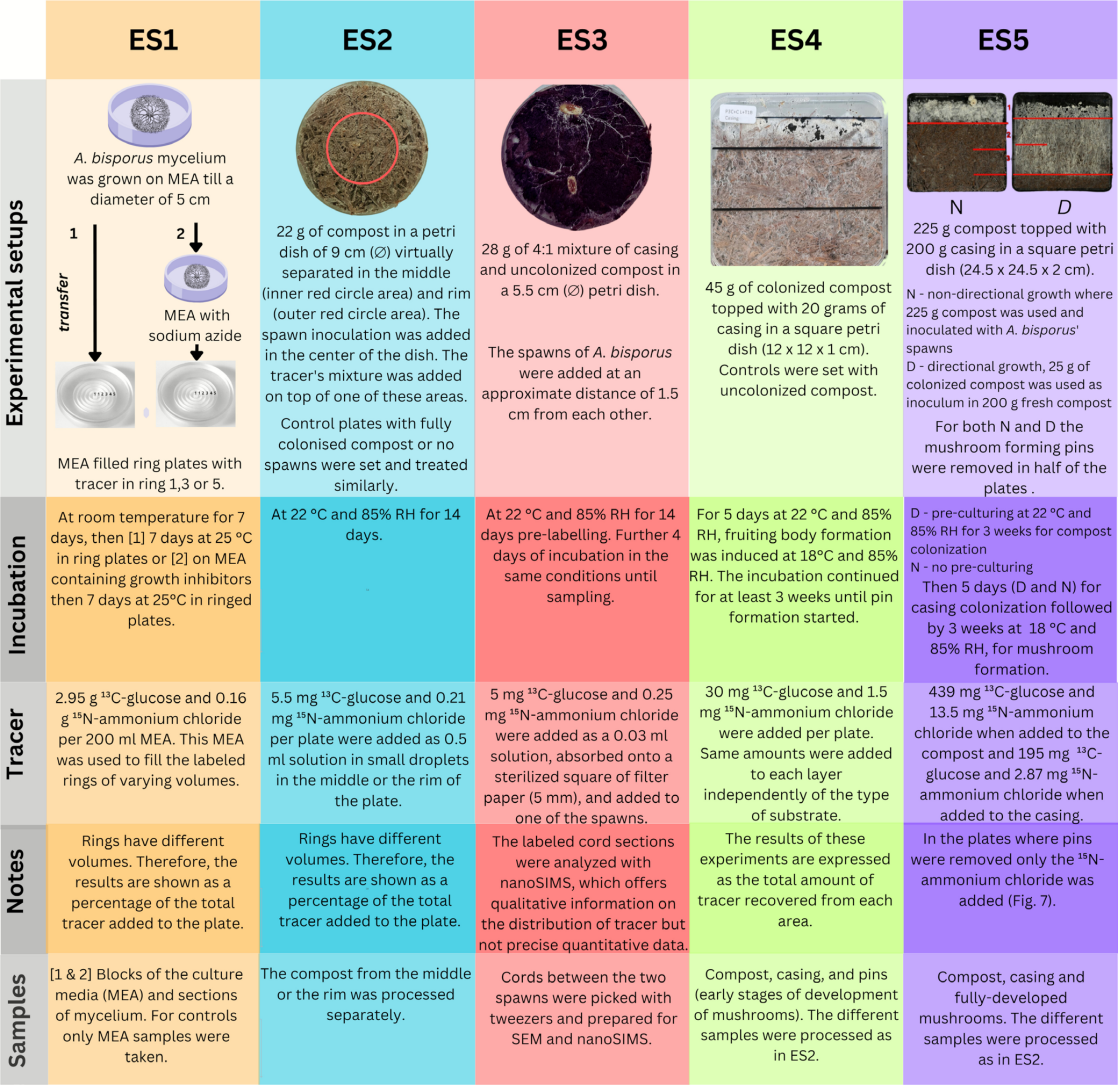
Schematic representation of ^13^C and ^15^N tracer‐based experimental setups.

*A. bisporus* strain A15 (Sylvan, the Netherlands) was used as model system throughout this study. The carbon and nitrogen translocation experiments were carried out using five distinct experimental setups (ES) (Figure 1, ES1 to ES5). Round Petri dishes (9 cm and 5.5 cm ø) were used for the axenic (ES1), compost (ES2) and fungal cords (ES3) specific nutrient translocation experiments. For assessing nutrient translocation in axenic cultures (ES1), pre‐grown colonies were prepared as follows. A polycarbonate track‐etched membrane (PCTE, 7.6 cm diameter, 0.1 μm pore size, Screening Devices, Amersfoort, the Netherlands) was placed on malt extract agar (MEA) (30 g/L malt extract, 5 g/L mycological peptone, 15 g/L agar, CM0057, Oxoid, Hants, United Kingdom) in 9 cm round Petri dishes. The membrane was inoculated with a mycelial plug and incubated for 7 days at 25°C. The pre‐grown colonies on membranes were then transferred as a whole onto custom‐made ring plates (Levin et al. 2007) (Table S1) containing MEA, with the tracer applied in either Ring 1, 3 or 5. These rings were chosen as representative as they coincide with the centre, middle and periphery of the colony. The plates were incubated at 25°C for an additional 7 days post‐transfer to allow tracer uptake, translocation and secretion.

In different experimental setups, compost (CNC Grondstoffen, Milsbeek, the Netherlands) and casing soil (Legro, Helmond, the Netherlands) served as growth substrates (ES2–ES5). The compositions of these substrates were previously detailed (Jurak (2015) for compost and Noble et al. (1999) for casing soil), but the quality of batches and the raw materials can vary. Round Petri dishes with 9 cm diameter were employed to investigate the influence of mycelial growth on nutrient translocation within the compost (ES2). In this set of experiments, tracers were applied to the middle or the rim of dishes filled with 22 g phase‐II end compost and inoculated with a spawn grain at the centre (Figure 1). The two areas, middle and rim, were not physically separated, but arbitrarily chosen, and their size was kept constant in all samples. As controls, the same methodology was applied to both the phase II‐end compost without spawn inoculation and the fully colonised compost (phase III‐end), where minimal to no fungal growth was anticipated in both instances. Tracers were applied only in the middle of the compost‐filled Petri dishes for these control setups (Figure 1). These dishes were then incubated for 14 days at 22°C and 85% relative humidity (RH). For cord formation experiments, a mixture of 1:4 casing to phase II‐end compost was utilised (ES3). The mixtures were introduced into 5.5 cm diameter round Petri dishes, followed by the addition of two rye spawn grains spaced approximately 1.5 cm apart. Incubation of these Petri dishes was carried out for 14 days at 22°C and 85% RH. Following labelling with stable isotope tracers (as outlined in subsequent sections), the plates were incubated for 3 days under the same conditions.

Experiments carried out in square plates (12 × 12 × 1 cm; Greiner, Alphen aan den Rijn, the Netherlands, or 24.5 × 24.5 × 2 cm; Nunc Square BioAssay Dishes, 10489282, Thermo Fisher Scientific, Wilmington, United States) represented small scale reconstructions of cross‐sections of the commercial compost bed. The smaller plates (ES4) were filled with 45 g phase III‐end compost and 20 g casing, and were used to test the translocation of both carbon and nitrogen during initial stages of cropping (casing colonisation and pinning) as well as their translocation in the absence of *A. bisporus*. The upscaled incubation in larger dishes (ES5) was performed to test transport during mushroom development using two different types of growth of *A. bisporus*: directional versus non‐directional growth (Herman et al. 2020). Directional growth involved the use of a culture consisting of a layer of phase III‐end compost (25 g) as inoculum, a layer of phase II‐end compost (200 g), and a layer of casing soil (200 g), while non‐directional growth used a substrate comprising a layer of phase III‐end compost (225 g) as inoculum and a layer of casing soil (200 g) (Figure 1, ES5). First, colonies were incubated for 5 days at 22°C and 85% RH to allow colonisation of the casing. Then, to induce fruiting body formation, the same cultures (ES5), were transferred to 18°C and 85% RH and further incubated for at least 3 weeks until 1 cm wide primordia developed. Then, isotope tracer was administered to the cultures which were further incubated for typically 3 days to allow outgrowth of the mushrooms.

### 2.2 Stable Isotope Labelling

Translocation of nutrients was studied using the stable isotope tracers ^13^C‐glucose (Campro Scientific, Veenendaal, the Netherlands; all 6 carbons were labelled) and ^15^N‐ammonium chloride (Campro Scientific) for carbon and nitrogen, respectively, as indicated (Figure 1). Water translocation was traced using deuterated water (D_2_O; Campro Scientific). In axenic cultures (ES1), the tracers were mixed with the agar medium by adding 2.95 g ^13^C‐glucose and 0.16 g ^15^N‐ammonium chloride per 200 mL MEA. For D_2_O, a labelling enrichment of *δ* = 1000‰ was aimed for in MEA. Tracer additions to experiments with compost or casing soil (ES2, ES4 and ES5) amounted to 5.5 mg ^13^C‐glucose and 0.21 mg ^15^N‐ammonium chloride per Petri dish in ES2, whereas 30 mg ^13^C‐glucose and 1.5 mg ^15^N‐ammonium chloride were added per 12 × 12 × 1 cm square plate in ES4. In ES5, 439 mg ^13^C‐glucose and 13.5 mg ^15^N‐ammonium chloride were added to the compost and 195 mg ^13^C‐glucose and 2.87 mg ^15^N‐ammonium chloride were added to the casing. In the experimental setups ES2, ES4 and ES5, the tracers were added as small droplets randomly distributed across the labelling area. In order to achieve sufficient enrichment, the required amounts of tracer were calculated according to De Kluijver et al. (2014) using the measured carbon and nitrogen content of the MEA, compost and casing by IRMS (see below). In cord studies (ES3), labelling with ^13^C‐glucose and ^15^N‐ammonium chloride was performed by applying sterilised squares (0.5 cm^2^) of filter paper (Whatman, Grade No. 1) on top of the rye spawn grain (inoculum), after which 5 mg ^13^C‐glucose and 0.25 mg ^15^N‐ammonium chloride dissolved in 25 μL of sterile MilliQ water were added. In summary, tracer enrichment was studied in the agar (ES1), axenic mycelium (ES1), bulk compost (ES2, ES4, ES5), mushroom pins (ES4), fully formed mushrooms (ES5) and cords (ES3) (Figure 1).

### 2.3 Transport of Stable Isotope Tracers in Axenic Colonies

In this experiment (ES1, Figure 1), 5 cm diameter *A. bisporus* colonies, pre‐grown on PC membranes, were directly transferred to MEA‐filled ring plates to investigate the effects of various processes—including evaporation/condensation, passive diffusion, wicking and growth‐induced mass flow—on nutrient translocation. Specific rings in these plates contained D_2_O, ^13^C‐labelled glucose, and/or ^15^N‐labelled ammonium chloride, where the stable isotopes contained in these compounds, D, ^13^C and ^15^N, were used as tracers. The colonies were incubated for 7 days at 25°C to allow for translocation of the isotope tracers.

Controls for this experiment included the following conditions: (i) MEA‐filled ring plates without a PC‐membrane, thus with rings directly exposed, to test for diffusion between the concentric wells (all tracers) and degassing/evaporation and subsequent condensation of the tracer (NH_3_ and D_2_O (g)); (ii) MEA‐filled ring plates covered with a PC membrane, to test for diffusion across the membrane; and (iii) MEA‐filled ring plates covered with a PC membrane covered with dead mycelium, to test for diffusion/wicking along the mycelium. Mycelium was killed by incubation for 3 h in a 60°C stove (Wuest et al. 1970). All these control conditions had either Ring 1 or 5 filled with MEA mixed with either deuterated water or both ^13^C‐glucose and ^15^N‐ammonium chloride, while the other rings contained unlabelled MEA.

After 7 days of incubation with isotope tracers, mycelium samples were collected by marking the rings on the fungal colony with a custom‐made stamp (imprinting the rings) and cutting out the mycelium rings using a scalpel. Mycelium from rings 1, 3 and 5 was transferred to 1.5 mL Eppendorf tubes, while the MEA in the ring plate of the corresponding rings was placed in 15 mL tubes. For the controls, only MEA samples were taken since no mycelium was present or the mycelium was dead. Samples from rings were freeze‐dried overnight and then disrupted at 25 Hz for 10 min using Tissuelyzer II (Retsch GmbH, Haan, Germany) for mycelium samples or a heavy‐duty paint shaker (SK550 1.1, Fast & Fluid, Sassenheim, Netherlands) for MEA samples. In both techniques, two 5 mm steel ball bearings (type 229503, Bofix, Wijk bij Duurstede, the Netherlands) were added to the tubes for disruption of the sample. MEA samples from deuterated water cultures were melted in a 90°C water bath and mixed 1:1 (vol:vol) with 20% sulphuric acid (H_2_SO_4_) to prevent solidification of the agar after subsequent cooling the samples to room temperature.

### 2.4 Cultivation‐Like Mesocosms

The initial 12 × 12 × 1 cm mesocosms were set up with and without *A. bisporus* (ES4, Figure 1) using plates filled with PII‐end and PIII‐end compost, respectively. The square plate culture was divided into three layers: the casing soil layer (1), the upper half of the compost layer (2), and the lower half of the compost layer (3). The isotope tracers were added to the different layers when the compost was topped with casing soil before the fungal network colonised the casing soil. The incubation was carried out at 22°C and 80% RH until day 7, after which pinning (primordia formation) was induced by venting at 18°C at 80% RH until day 25. At the end of the incubation all the layers and pins bigger than 5 mm were collected.

In the upscaled set‐up (ES5, Figure 1), two types of cultures were used: those where pins were continuously removed to study transport within the vegetative mycelium, and those where pins were allowed to develop into mushrooms, to study nutrient transport to the mushrooms. In the plates where pins were removed, a single layer was labelled by adding 2 mL of ^15^N‐ammonium chloride dissolved in water. In the plates where pins were not removed, a single layer was labelled by adding 2 mL of ^13^C‐glucose and ^15^N‐ammonium chloride dissolved in water. All the plates were incubated for 3 days at 18°C and 85% relative humidity until the mushrooms fully developed (Hammond and Nichols 1976). To determine the actual initial tracer enrichment value obtained per layer, square plate cultures were labelled in all layers simultaneously with 2 mL ^15^N‐ammonium chloride dissolved in water per layer and incubated for 3 h at 18°C and 85% humidity. Each layer was sampled separately and stored in plastic bags. This incubation period was necessary to ensure that the tracer (^15^N‐ammonium chloride) was properly absorbed by the substrate.

At the end of the incubations (ES4 and ES5), all three layers were sampled and freeze‐dried for 2 days and subsequently disrupted for 1.5 min using a HP‐MA automatic pulverising mill in a tungsten‐carbide grinding vessel (Herzog Maschinenfabrik GmbH & Co., Osnabrueck, Germany). The samples were then analysed using bulk IRMS (Thermo Delta V). Pin (ES4) and mushroom (ES5) samples were also taken to study how much isotope tracer they incorporated from each layer (see Equation S1) and processed in a similar fashion to layer samples.

### 2.5 Water Uptake Requirement for Translocation

As an alternative to deuterated water experiments, the requirement for water uptake to induce translocation was studied by evaluating the uptake and translocation of deoxy‐glucose labelled with an infrared fluorescent dye diluted in water (Bleichrodt and Wösten 2022) as follows. *A. bisporus* colonies pre‐grown on MEA and measuring 5 cm in diameter were transferred to ring plates filled with MEA (as in ES1, Figure 1). To prevent the colony centre from accessing water, the central ring (Ring 1) was left empty. Alternatively, all rings were filled with MEA as a control. A 6‐mm disk of Whatman filter (Grade no. 1) paper was placed at the centre of the colonies, onto which 5 μL of 2‐deoxy‐D‐glucose labelled with an infrared fluorescent dye (IRDye 800CW 2‐DG Optical Probe, LI‐COR Biosciences, Lincoln, United States) was added at a concentration of 100 ng/mL. The colonies were then incubated for 7 days at 25°C. After incubation, uptake and translocation of IRDye was examined by analysing fluorescence using an Odyssey CLx imaging system (emission = 800 nm, scan mode = auto gain, pixel size = 337 μm, focus depth = 4 mm, LI‐COR Biosciences, Lincoln, United States).

### 2.6 Isotopic Ratio Mass Spectrometry

For bulk IRMS analysis of samples from experiments ES1, ES2, ES4 and ES5 (Figure 1), 2.0–2.5 mg of sample was weighed and combusted. The following standards were used: 0.75–1.00 mg IAEA‐CH‐7 (International Atomic Energy Agency) for the isotopic signature of carbon, 0.20–0.25 mg Ammonium Sulphate Standard (ASS) (Merck), for the isotopic signature of nitrogen, and 2.0–2.5 mg Isotopenstandard Weizenmehl, zertifiziert (IVA Analysentechnik) which closely resembles the wheat‐straw based compost, for both carbon and nitrogen quantification and isotopic signature verification. Samples and standards were weighed in tin capsules (5 × 9 mm, IVA Analysentechnik) and measured with a Flash IRMS elemental analyser (Thermo Scientific) coupled to a Delta V Advantage IRMS (Thermo Scientific).

Phospholipid derived fatty acids (PLFAs) extractions were performed from the same dry compost samples used in the bulk analysis using the protocol of De Kluijver et al. (2014) and measured for hydrogen (D) isotope enrichment with a GC‐TC‐IRMS (Agilent 8890 coupled to an Elementar GC5 and an Elementar Isoprime vision) with an apolar column (J&W CP‐Sil 5 CB GC Column, 25 m, 0.32 mm, 0.40 μm). The standard *n*‐alkane A3 mix (by Arndt Schimmelmann, Indiana University) was used for the isotopic signature of both carbon and hydrogen.

For D_2_O experiments in ring plates (similar to ES1; Table S2), agar samples from rings were harvested and boiled in 15 mL tubes (Greiner) and 1 volume 19.2% sulphuric acid was added to prevent solidification of the agar. In 12 mL Exetainer vials (Labco) 200 μL sample was pipetted and a platinum catalyst was inserted. The vials were flushed with 3% H_2_ in Helium. After 40 min the gases in the headspace were measured with a GasBench II (Thermo Scientific) coupled to a Delta V Advantage IRMS (Thermo Scientific).

Isotope enrichment is expressed using the delta (δ) notation and reported relative to Vienna Standard Mean Ocean Water (VSMOW, for δD); Vienna Pee Dee Belemnite (VPDB, for δ^13^C), and air (for δ^15^N).

### 2.7 Cords Preparation and nanoSIMS Imaging

The fungal cords obtained in dedicated experiments (ES3, Figure 1) were picked with tweezers, washed with PBS, and fixed in a solution of 2.5% glutaraldehyde (Aqueous Glutaraldehyde EM Grade 8%, EMS) and 4% paraformaldehyde (8% Paraformaldehyde Aqueous Solution, EM Grade, EMS) at 4°C for 48 h to preserve the protein and polysaccharide structures of the cells. Afterwards, the filaments were post‐fixed in 1% osmium tetroxide (from a stock of osmium tetroxide 4% sol., EMS) in double distilled H_2_O to stabilise the lipidic organelles of fungal and bacterial cells. Subsequently, the samples were dehydrated using an ethanol gradient at 4°C (Fixation, n.d.; Glauert 1975). The final solution of ethanol was drained as much as possible and the cords were immersed in a mixture of acetone:EPON‐araldite resin (Araldite/Embed kit, EMS) as described in the manufacturer's protocol. This mixture varied in proportion from 2:1 until pure EPON‐araldite resin. Polymerisation was induced with the addition of benzyldimethylamine (BDMA, EMS). The resin was cut in thin slices perpendicular to the axis of the cord, using an ultramicrotome (Leica). The thin slices of 375 nm were deposited on silicon wafers using a heated plate at 40°C. These cord pre‐treatment procedures were needed for optimal imaging, more specifically they ensured the sample was well preserved and flat. However, they introduced additional carbon from the resin, diluting the ^13^C labelling of the sample.

Samples were imaged using an optical microscope, SEM (ZEISS GeminiSEM), and finally with a NanoSIMS 50 L instrument to image isotopic composition of the samples on a single‐cell level. For the latter, the imaged secondary ions included ^16^O^−^, ^12^C_2_^−^, ^12^C^13^C^−^, ^12^C^14^N^−^, ^12^C^15^N^−^, ^31^P^−^ and ^32^S^−^. NanoSIMS data processing and analysis were done using the Look@NanoSIMS software (Polerecky et al. 2012). First, the secondary ion images were aligned and accumulated. Then, regions of interest (ROI) corresponding to hyphal cells (cross‐sections) were drawn manually using the overlay between the ^12^C_2_^−^, ^12^C^14^N^−^ and ^31^P^−^ signals and classified based on their morphology as ‘membrane‐rich’ (high content of inner‐membrane structures) and ‘void’ (low counts and no membrane structures in their lumen). Finally, ROI‐specific isotope ratios ^13^C/^12^C and ^15^N/^14^N were calculated according to 0.5 × ^12^C^13^C^−^/^12^C_2_^−^ and ^12^C^15^N^−^/^12^C^14^N^−^, respectively, using the total ion counts accumulated over the ROI pixels. The single‐cell level ^13^C and ^15^N isotope enrichments are reported in the δ notation relative to control samples with a natural ^13^C and ^15^N isotope composition.

### 2.8 Statistical Analysis

For comparing means, independent Student's *t*‐tests or ANOVAs with Tukey or LSD post hoc tests were performed. NanoSIMS data was analysed with Look@NanoSIMS software (Polerecky et al. 2012) and Pearson correlation analysis was performed. *p* < 0.05 was considered as significant. Specific statistical tests are mentioned in the legends for each figure and significant effects are indicated within figures.

## 3 Results

### 3.1 Directionality and Quantification of Translocation in Vegetative Mycelium

We set out to investigate how different parts of the fungal network take up and transport nutrients. Our hypothesis was that both carbon and nitrogen would be taken up throughout the colony and transported to the colony margin via growth induced cytoplasmic bulk flow (Heaton et al. 2010). To this end, *A. bisporus* was pre‐grown on porous polycarbonate (PC) membranes for 7 days and then transferred for 7 days to MEA filled ring plates of which specific rings were labelled with both isotope tracers ^15^N‐ammonium chloride and ^13^C‐glucose (Figure 1, ES1). Ring plates were used to minimise translocation of tracers between different medium rings by diffusion, since the walls defining the rings physically separate the rings of medium. Both mycelium and culture medium were sampled for analysis.

**FIGURE 2:**
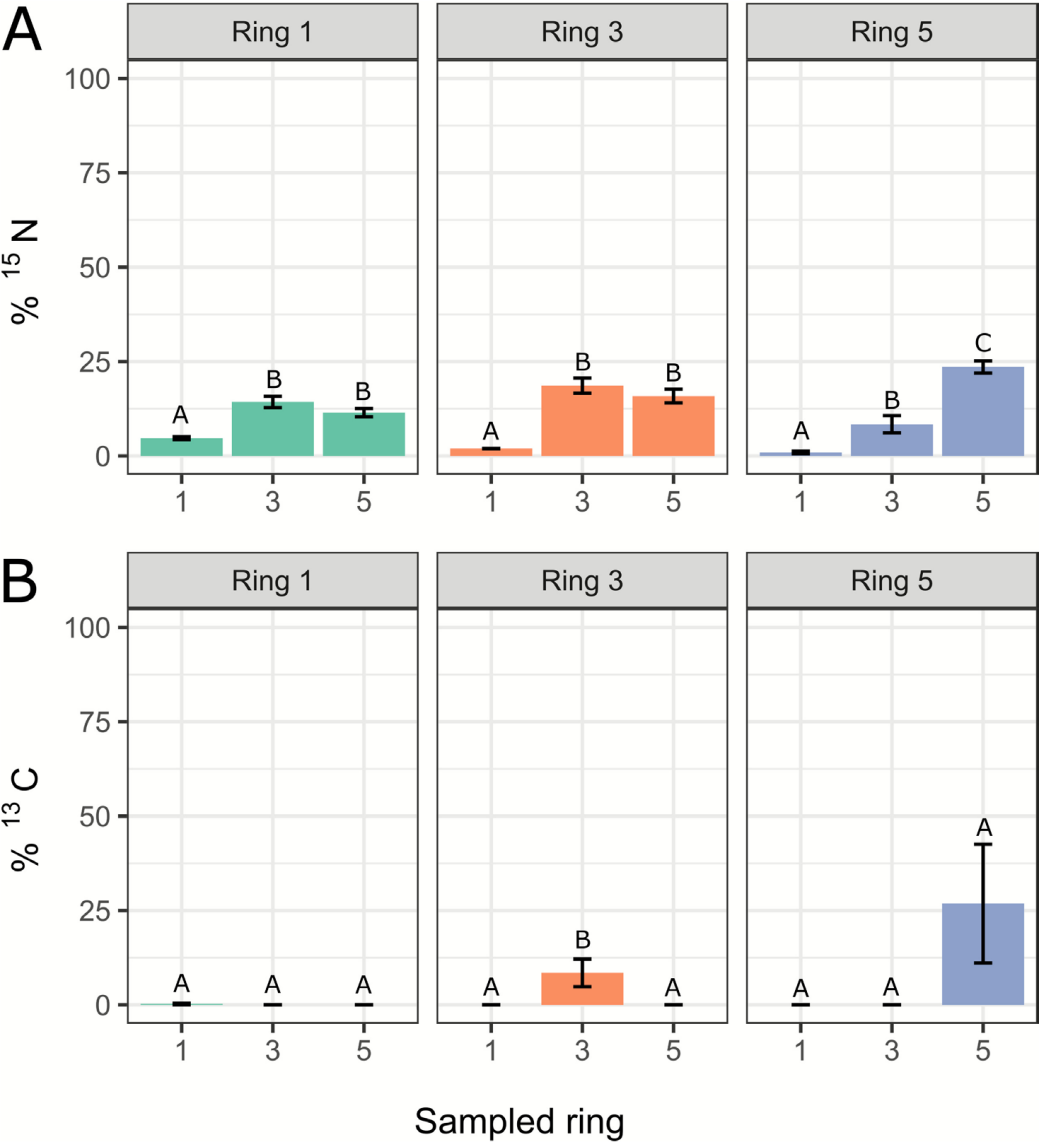
Enrichment of MEA samples that had been incubated with living fungus for 7 days. *X*‐axis shows the sampled ring, *Y*‐axis shows tracer found as a percentage of total tracer added to the plate. (A) The ^15^N content of the sampled rings; (B) the ^13^C content of the sampled rings. The graph titles, Ring 1, Ring 3 and Ring 5, indicate the respective areas where the ^13^C‐glucose and ^15^N‐ammonium chloride were initially added. Error bars represent standard deviations (SD) (*n* = 3). One‐way ANOVA with Tukey post hoc test was performed, comparing tracer enrichment between rings when one ring was labelled.

**FIGURE 3:**
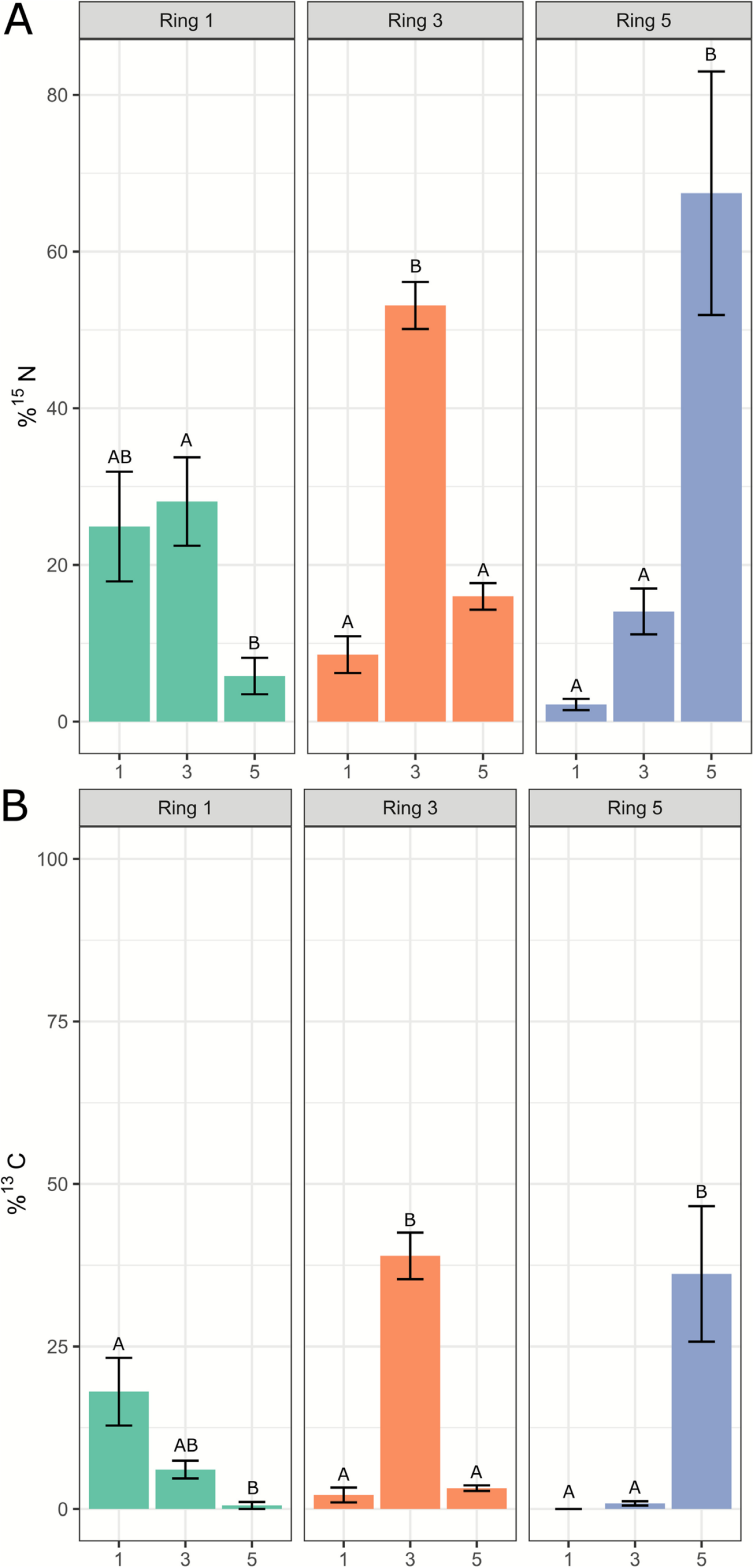
Enrichment of mycelium samples from plates where different rings had been labelled. *X*‐axis shows the sampled ring, *Y*‐axis shows tracer found as a percentage of total tracer added to plate. (A) ^15^N enrichment in plates where Ring 1, 3 or 5 was labelled. (B) ^13^C enrichment in plates where Ring 1, 3 or 5 was labelled. The titles of each graph indicate the labelled ring (1, 3 or 5). Error bars represent standard deviations (SD) (*n* ≥ 3). One‐way ANOVA with Tukey post hoc test was performed, comparing tracer enrichment between rings when one ring was labelled.

After incubation with living fungus, the fungus was removed from the ring plates and MEA was harvested from Ring 1, 3 and 5. Thereafter, ^13^C and ^15^N enrichment in MEA samples was analysed with bulk IRMS. ^15^N was detected in the MEA of all sampled rings (Figure 2A), suggesting potential nitrogen uptake by the mycelium, translocation, and subsequent secretion or exudation into the medium. Nitrogen enrichment in rings distant from the labelled ring occurred both towards the centre and the periphery (Figure 2A), but more pronounced in the direction of the periphery. Living mycelium samples showed a similar trend (Figure 3A). Notably, relatively more tracer was detected in the fungal biomass than in the underlying medium in the respective rings (Figures 2A and 3A), indicating uptake and potential incorporation. Overall, there was relatively greater ^15^N translocation to the adjacent rings (Figure 3A) compared to ^13^C (Figure 3B), most likely because ^13^C is lost from the culture due to respiration by the fungus, which is not the case for ^15^N.

For glucose‐derived ^13^C from plates with living fungus, the results were different. ^13^C enrichment in MEA was not detectable in rings to which no isotope tracer had been added (Figure 2B). The quantity of ^13^C still present in the labelled ring varied depending on the location where the tracer was added. When ^13^C‐glucose was added to the centre (Ring 1), no ^13^C enrichment was found in this ring, suggesting complete uptake (Figure 2B, Ring 1). This indicates that there was no translocation of the tracer by the mycelium and subsequent secretion into rings other than the labelled one. When rings 3 and 5 were labelled, respectively, ^13^C enrichment in the MEA was higher in Ring 5 than in Ring 3 (Figure 2B). This may be due to a lower density of mycelium growing on top of Ring 5 than on Ring 3, resulting in less uptake. Living mycelium samples exhibited a ^13^C enrichment pattern where the labelled ring generally had the highest enrichment, and subsequent rings showed progressively lower enrichments, indicating some translocation by the fungus (Figure 3B). By and large, tracers were preferentially translocated towards the colony margin. However, some translocation was also detected towards the colony centre. The lack of ^13^C secretion into the MEA, indicates that glucose is used mainly locally for energy production purposes by the mycelium. Little of the glucose‐derived carbon is transported and used to synthetise proteins, which are expected to be abundant in the exudates of *A. bisporus*. On the other hand, the areas to which ^15^N was transported could primarily have taken up unlabelled carbon locally to build secreted proteins, which could explain the above observations.

In the above experiments we looked at total translocation and did not focus on the underlying mechanisms. Therefore, we next aimed to dissect the mechanisms of this translocation by performing carefully designed control experiments (Figure [Link], [Link]). As controls, we checked for translocation in ring plates filled with MEA that was (i) not topped, (ii) topped with a PC membrane, or (iii) topped with a PC membrane with a dead fungal colony. Only the culture medium was sampled in these controls. The first two treatments were included to test for translocation between the medium filled rings and across the membrane, respectively. This could theoretically happen by diffusion (if medium of adjacent rings was slightly in contact) or by tracer degassing (NH_3_) and subsequent condensation (NH_3_)/sequestration (NH4^+^). The treatment by killing the fungus was performed to check the impact of fungal live on translocation. In case of this treatment, theoretically translocation could additionally happen through intracellular/extracellular diffusion or external wicking along the hyphae (Read and Stribley 1975). However, we cannot exclude that there may be other yet unknown potential underlying processes.

Uncovered MEA‐filled rings (condition i) exhibited small but detectable ^15^N translocation, but did not show ^13^C translocation (Figure S1A,D). The latter suggests that there is no diffusion of ^13^C tracer between the agar medium in adjacent rings. The ^15^N translocation was likely due to the conversion of ammonium to ammonia in the labelled ring, followed by degassing, translocation through the headspace, and subsequent dissolution into other rings. The presence of a PC membrane (condition ii) resulted in ammonium‐derived ^15^N translocation, but limited glucose‐derived ^13^C translocation (Figure S2B,E). This indicates that diffusion of tracers across the membrane and into the medium is happening. Next, we killed colonies with heat (condition iii) (Figure S1C,F). MEA collected from these plates showed ^15^N and ^13^C enrichment in all sampled rings (Figure S1C,F). Moreover, the translocation of ammonium derived ^15^N in MEA towards the colony margin was higher when the fungus was killed (Figure S1F) (condition iii) compared to the results with living mycelium (Figure 2). For ^13^C‐glucose translocation both conditions were quite similar, except that less tracer was gone from the central ring in case of the killed fungus (Figure S1F, Ring 1) (condition iii). Notably, enrichment for both ^15^N and ^13^C was observed away from the labelled ring, particularly towards the centre (Figure S1C,F) (condition iii). In this condition, the translocation of both tracers in the direction of the colony margin was higher than in control condition ii (Figure S1B,E), indication that wicking or intra−/extracellular diffusion along the fungal network is happening.

**FIGURE 4:**
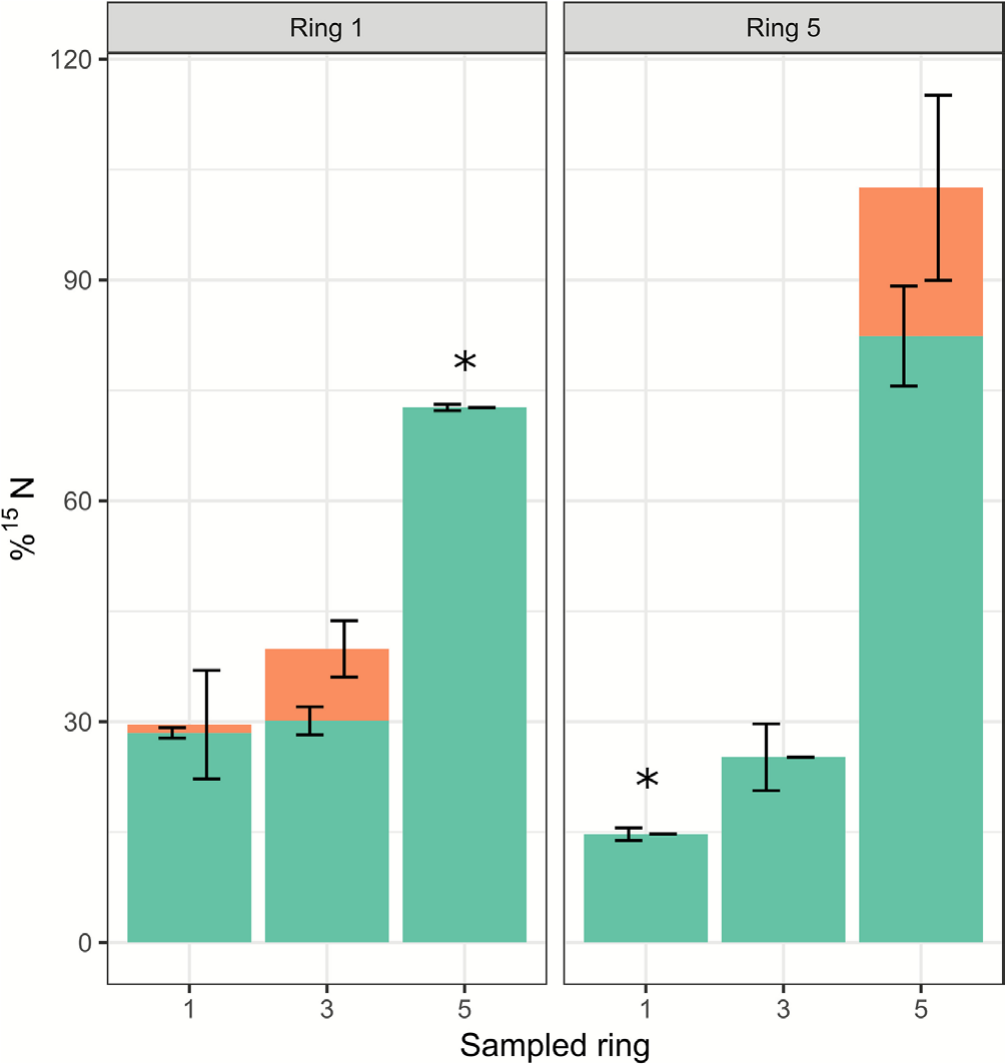
Distribution of average ^15^N enrichment across sampled rings showing background translocation (control conditions; green bars) versus uptake and transported tracer contributions (orange bars). ‘Above background’ (orange bars) data combines MEA (Figure S1) and mycelium samples (Figure 3), while ‘background’ (green bars) data is derived exclusively from MEA samples (Figure S1). The *X*‐axis denotes the sampled ring, and the *Y*‐axis shows tracer content as a percentage of the total added to the plate. The left panel presents mean values for plates labelled at Ring 1, and the right panel displays mean values for plates labelled at Ring 5 (graph titles). Error bars represent SD (*n* = 3). Student's *t*‐tests were performed for each sampled ring comparing above background and background levels. Significant effects are indicated with asterisks.

By and large, the observed ^13^C translocation in case of living fungus (Figures 2 and 3) was explained by passive processes that were assessed in the control conditions (Figure S1). In contrast, a small part of the observed ^15^N translocation in the direction of the colony margin (Figure 4) was explained by active processes such as fungal growth (see also Figure 2). This indicates that growth is not the major driver for the observed translocation in these experiments.

### 3.2 Water Uptake Is Required to Drive Nutrient Translocation in Axenic Conditions

We attempted to assess water translocation by *A. bisporus* using deuterated water. However, translocation of deuterated water showed high background levels in the absence of *A. bisporus* in ring plate experiments (ES1, Figure 1 and Table S2). In contrast, glucose translocation under similar conditions was minimal (Figure S1). This suggests that the water moved through evaporation from the labelled ring and subsequently condensed in the unlabelled rings (Table S2). This prevents the use of deuterium oxide as a reliable tracer for intercellular water transport by the mycelium. High variability in deuterium enrichment was also observed in compost incubations (ES2; Figure S2A). Moreover, this variability did not only occur in compost enriched with deuterium, but also in deuterium enrichment of PLFA biomarkers for bacteria (data not shown) and fungi (Figure S2B). The high variability of deuterium tracer studies made it impossible to use this tracer quantitatively to assess water transport. However, it shows that water can quickly move through cultures by evaporation and condensation.

**FIGURE 5:**
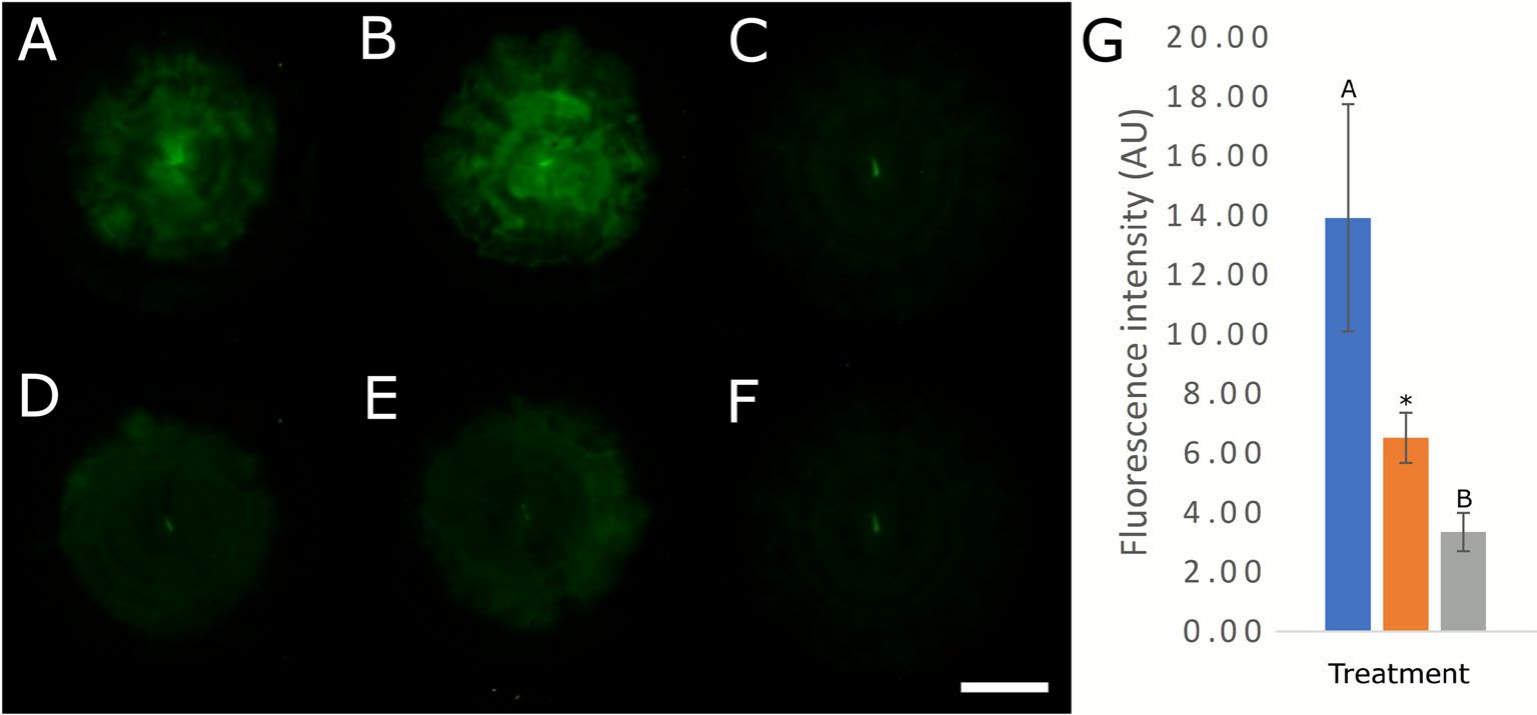
Fluorescence images of IRDye 2‐deoxy‐D‐glucose translocation after a 7 days incubation period in *A. bisporus* axenic cultures on MEA. Control plates (A–C) had the first ring filled with MEA, providing access to water, while experimental plates (D–F) had the first ring left empty, restricting water access in this region, while all remaining rings contained MEA. The tracer was applied to the centre of the colonies on day 1 (A, B, D, E). Plates (C, F) served as controls without IRDye to account for background autofluorescence. The inoculum, which is autofluorescent, can be seen as small fluorescent rod in the centre. The scale bar represents 28 mm. (G) Mean fluorescence intensities ± SD of IRDye 2‐deoxy‐D‐glucose in colonies (A, B) blue bars, (D, E) orange bars, (C, F) grey bars. ANOVA with Tukey post hoc test was performed, and showed a trend (‘*’, *p* = 0.095) between blue and orange bars, while LSD post hoc test showed a significant difference (‘*’, *p* = 0.048).

To test whether local water uptake is required to drive nutrient translocation, we transferred *A. bisporus* colonies on a membrane to MEA filled ring plates (see ES1 setup; Figure 1) of which the centre (Ring 1) was either deprived of water (not filled with MEA) or not (contained MEA). Subsequently, the mycelium on top of Ring 1 was labelled with non‐metabolizable fluorescently labelled 2‐deoxy‐D‐glucose to follow translocation. Fluorescence imaging revealed translocation of the nutrient in control plates that had access to water in the colony (Figure 5A,B). In contrast, almost no nutrient translocation was observed when the colony centre had been deprived of water (Figure 5D,E). When corrected for the autofluorescence, translocation in the presence of water resulted in more than 3× higher tracer intensity (compare Figure 5A,B with Figure 5D,E, see also Figure 5G). Thus, the results suggest that local water uptake is essential for driving nutrient translocation.

### 3.3 Transport in Compost

**FIGURE 6:**
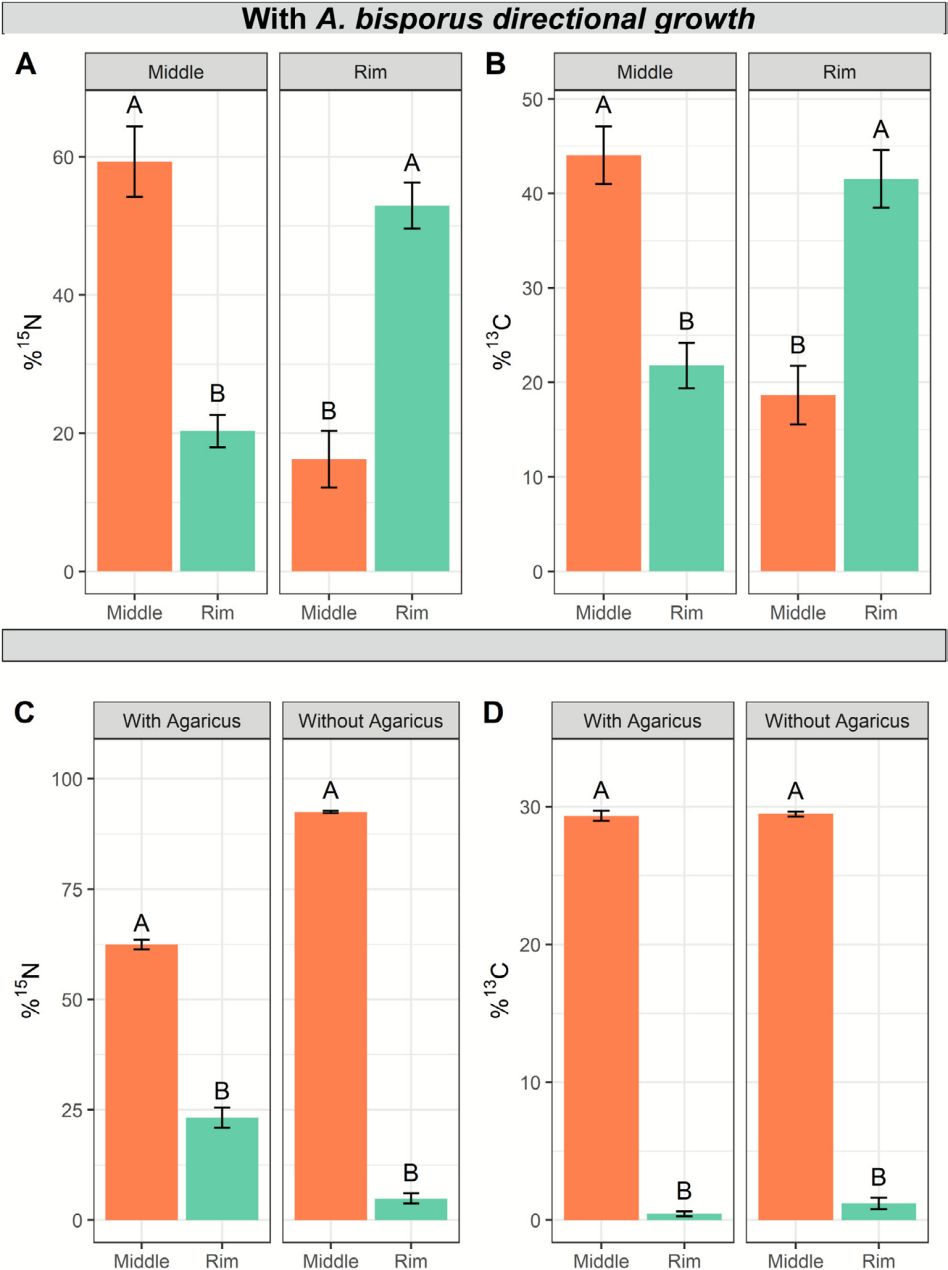
Distribution of tracer‐derived ^15^N and ^13^C in compost‐filled dishes. (A) The percentage of tracer‐derived ^15^N (A) or ^13^C (B) after the directional growth of *A. bisporus* in compost. In these conditions, at day 1, phase II‐end compost was inoculated with a spawn grain of *A. bisporus* in the centre of a Petri dish. The terms ‘Middle’ and ‘Rim’ in the graph titles indicate the locations where the tracers were originally introduced at day 1. Error bars represent standard deviation (SD) (*n* = 3). (C) ^15^N and (D) ^13^C translocation in phase II‐end compost (without *A. bisporus*) and in phase III‐end compost (with *A. bisporus*, non‐directional growth). In these conditions, the tracer was added to the middle of the plate and samples were taken in the Middle (orange bars) and in the Rim (green bars) as in ES2. Error bars represent the min and max values. The percentages indicate the proportion of tracer content found in each sampled area relative to the total amount initially added to the plate. The *X*‐axis represents the sampled areas after 14 days incubation. Student's *t*‐tests were performed, comparing tracer enrichment between middle and rim within cultures (indicated with letters), when either the middle or rim was labelled, and between conditions (with or without *A. bisporus*; no significant differences were found).

For industrial mushroom production, *A. bisporus* is commonly allowed to colonise compost that is later on topped with casing soil to induce mushroom formation. Therefore, we tested how tracers translocated in the presence or absence of *A. bisporus* in compost. To this end, PII‐end (uncolonized) and PIII‐end (16 days homogeneously colonised; no localised nutrient sink) compost were placed in Petri dishes as control for the absence and presence of mycelium, but the absence of directional growth, respectively (ES2, Figure 1). Alternatively, PII compost filled dishes were inoculated at the centre with a rye spawn grain to generate a directionally growing colony. The resulting radial extension in the colony margin acts as a nutrient sink (Herman et al. 2020). Immediately after filling, the centre or the periphery of the plate was labelled with both ^15^N‐ammonium chloride and ^13^C‐glucose. The central and peripheral zones of the compost (ES2, Figure 1) were sampled after 14 days and subjected to IRMS. In contrast to axenic conditions, isotopic measurements of the compost revealed similar translocation of tracer in both directions for either ammonium‐derived ^15^N or glucose‐derived ^13^C in directionally grown colonies (Figure 6A,B). Despite this bi‐directional nutrient movement, the mycelium of *A. bisporus* visibly expanded in one direction, that is, from the centre to the rim during the incubation period (PII compost + spawn). In cultures where *A. bisporus* was non‐directionally pre‐grown (homogeneously mixed spawn in PIII compost) prior to labelling, ^15^N translocated towards the rim of dishes in similar quantities as directional growth (Figure 6C). However, ^13^C translocated in relatively much lower quantities (Figure 6D). In incubations where *A. bisporus* was absent (PII‐end compost), minimal to no translocation of these elements occurred (Figure 6C,D), highlighting the influence of *A. bisporus* on nutrient translocation in the compost. Altogether, this shows that the presence of *A. bisporus* enhances tracer translocation, but in its absence some tracer translocation is also observed.

### 3.4 Transport of Nutrients in the Mushroom Bed

**FIGURE 7:**
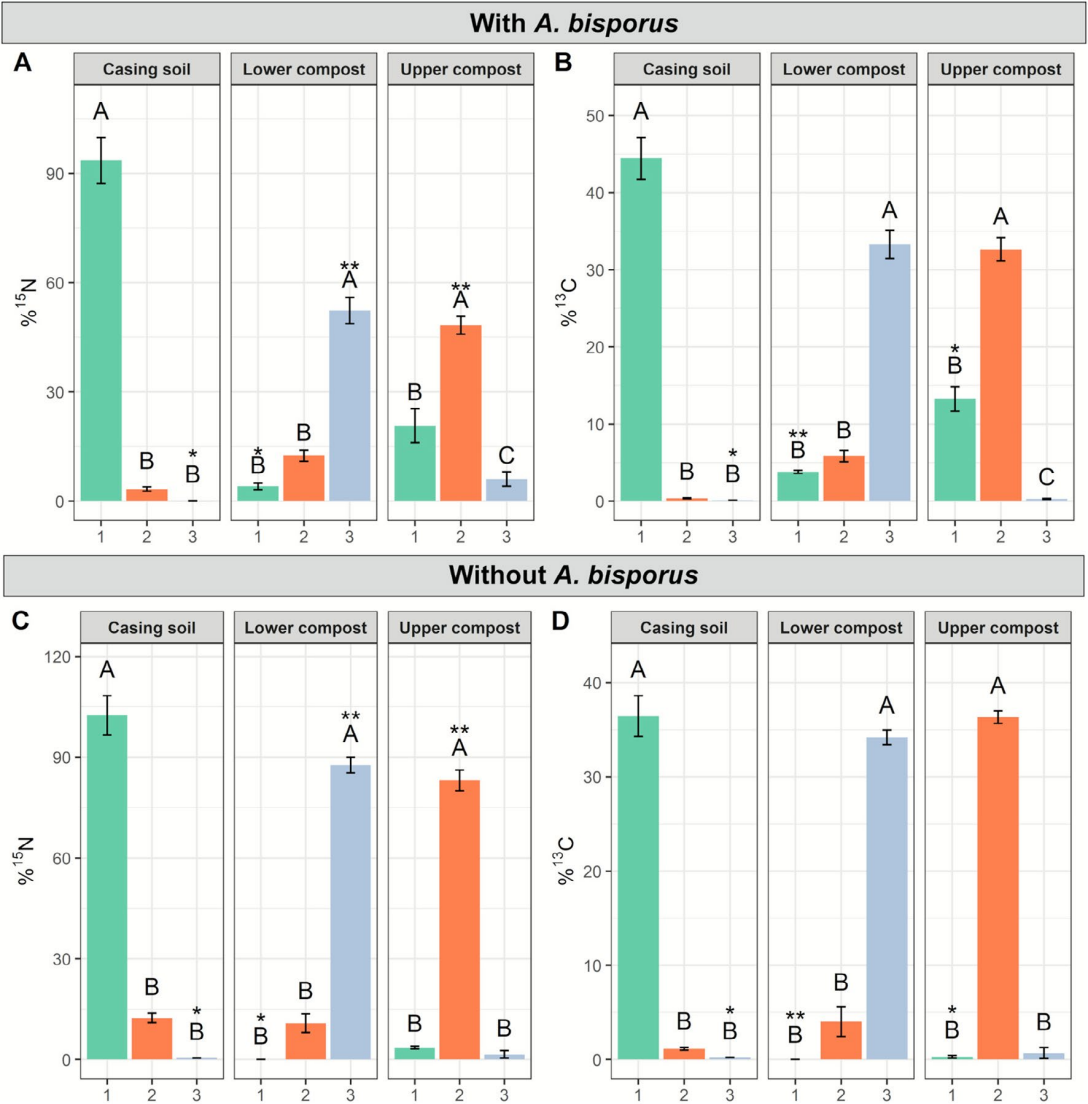
Tracer‐derived ^15^N and ^13^C translocation in the compost bed. Translocation of ammonium‐derived ^15^N (A, C) and glucose‐derived ^13^C (B, D) in the presence of *A. bisporus* (A, B; PIII‐end compost) or in the absence of *A. bisporus* (C, D; PII‐end compost). The sampling area number on the *X*‐axis indicates from which layer the samples were taken, with 1 for casing (green bars), 2 for upper compost (orange bars) and 3 for lower compost layers (blue bars). The headers above the panels indicate to which layer the tracer was added at *t* = 0 in the system. Error bars represent SD (*n* = 3). One‐way ANOVA with Tukey post hoc test was performed, comparing tracer enrichment between layers when one layer was labelled (indicated with letters). Student's *t*‐tests were performed to compare the same layer with and without *A. bisporus* (panel A vs. C and B vs. D), indicated with asterisks: *p* ≤ 0.001 ~ ***, *p* ≤ 0.01 ~ **, *p* ≤ 0.05 ~ *.

In an effort to replicate conditions resembling commercial cultivation, microcosms representing a cross‐sectional view of the mushroom cultivation bed were employed. To this end, square plates were either filled with PII‐end compost without *A. bisporus* or with colonised PIII‐end compost, abutted to a layer of casing soil. Cultures were incubated for 30 days, which coincides with the length of a commercial cropping cycle when two mushroom flushes are harvested (Figure 1, ES4). In the presence of *A. bisporus*, ^15^N and ^13^C tracers predominantly translocated from the compost towards the casing where mycelium was actively growing (Figure 7A,B). This movement occurred at similar relative rates in the compost as the average tracer‐derived carbon to nitrogen ratio 15:1 aligned with the average total carbon to nitrogen ratio of 15:1 as analysed by IRMS. The same trend was present in the casing, where the average carbon to total nitrogen ratio was 29.7 and 31 for the tracer‐derived nitrogen and carbon. Moreover, the upper compost layer contributed more in feeding the growing mycelium in the casing than the lower compost layer for both tracers (Figure 7A,B). Conversely, when the lower and upper compost layers were labelled, minimal to negligible translocation of both tracers was observed to the casing when *A. bisporus* was not present (Figure 7C,D). Moreover, there was no clear preference for translocation to layers above or below the labelled upper compost layer, indicating that any translocation observed was likely the result of random diffusion of the tracer or wicking along the solid matter particles enveloped in a water film, comprising the compost (Figure 7C,D). These findings suggest that *A. bisporus* mycelium primarily facilitates tracer translocation within the compost bed. However, when the tracer was added to the lower compost layer, a consistent presence of ^15^N and ^13^C in the upper compost layer was detected across treatments, with and without *A. bisporus*. Furthermore, no clear translocation from the upper compost layer to the casing was observed in the absence of *A. bisporus* (Figure 7C,D). This implies that some tracer can move within the compost but not from the compost to the casing or from the upper to the lower compost layer in the absence of *A. bisporus*, emphasising the role of *A. bisporus* in directing nutrient transport towards the casing layer where it actively grows. However, the tracer detected in adjacent layers to the labelled one in the absence of *A. bisporus* could also be attributed to low levels of cross‐contamination during sampling, as some labelled substrate might have been inadvertently exchanged while separating the layers.

**FIGURE 8:**
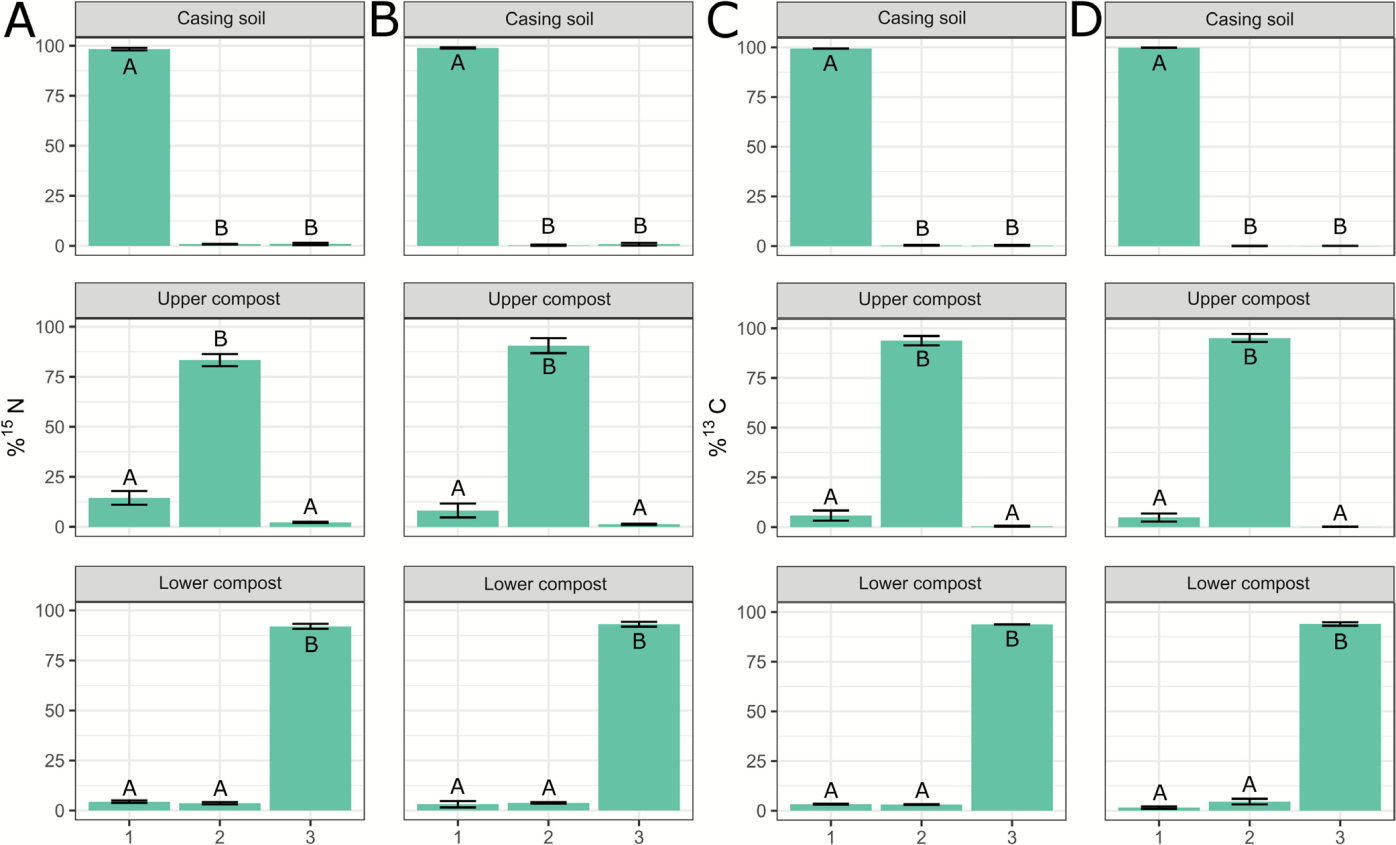
Translocation of (A, B) ^15^N and (C, D) ^13^C between different substrate layers in (A, C) directional and (B, D) non‐directional cultures. In directional cultures, a thin layer of phase III‐end compost was used as inoculum abutted to a large layer of PII‐end compost, whereas in non‐directional cultures, spawn was homogeneously mixed with phase II‐end compost to inoculate the substrate. The headers indicate which layer was labelled. The *x*‐axis indicates which layer was sampled (1: Casing soil, 2: Upper compost, 3: Lower compost), and the *y*‐axis shows the percentage of tracer detected relative to the total tracer added. Mushrooms were allowed to form during the experiment. Error bars represent standard deviation (SD) based on three replicates (*n* = 3). One‐way ANOVA with Tukey post hoc test was performed, comparing tracer enrichment between layers when one layer was labelled.

We investigated whether the network architecture impacts ^15^N and ^13^C translocation between different layers within the substrate during mushroom formation. To this end, larger mesocosms were established to allow for the development of fully formed fruiting bodies (ES5, Figure 1). Either the casing layer, top compost, or bottom compost layer were labelled using ^15^N‐ammonium chloride and ^13^C‐glucose. The results demonstrated enrichment of these labelled elements predominantly in the layers where the tracer was initially applied (Figure 8). In the case of ^15^N labelling (Figure 8A,B), labelled layers contained most of the tracer in the originally labelled layers. In the case of ^13^C labelling (Figure 8C,D), hardly any tracer translocated from casing to the compost. When the top compost or bottom compost layers were labelled, some ^13^C translocated to the casing layer and to the top compost layer, but almost not to the bottom compost layer. Relative more ^15^N translocated from each labelled layer to all other layers compared to ^13^C, albeit in small amounts. There were no substantial differences between directional (Figure 8A,C) and non‐directional growth (Figure 8B,D), thus, the network architecture does not impact nutrient translocation between substrate layers under these conditions.

Next we tested whether mushroom development impacts tracer translocation between layers. To this end, cultures were either grown directionally or non‐directionally and labelled with ^15^N‐ammonium chloride. To prevent mushroom development, mushroom primordia (1–3 mm) were directly picked after their formation. In other cultures, mushrooms were allowed to mature. Data indicate (Figure S3) that there are no major differences in ^15^N translocation between substrate layers. This shows that mushroom formation does not impact ^15^N tracer translocation between substrate layers.

### 3.5 Feeding of the Developing Mushrooms

**FIGURE 9:**
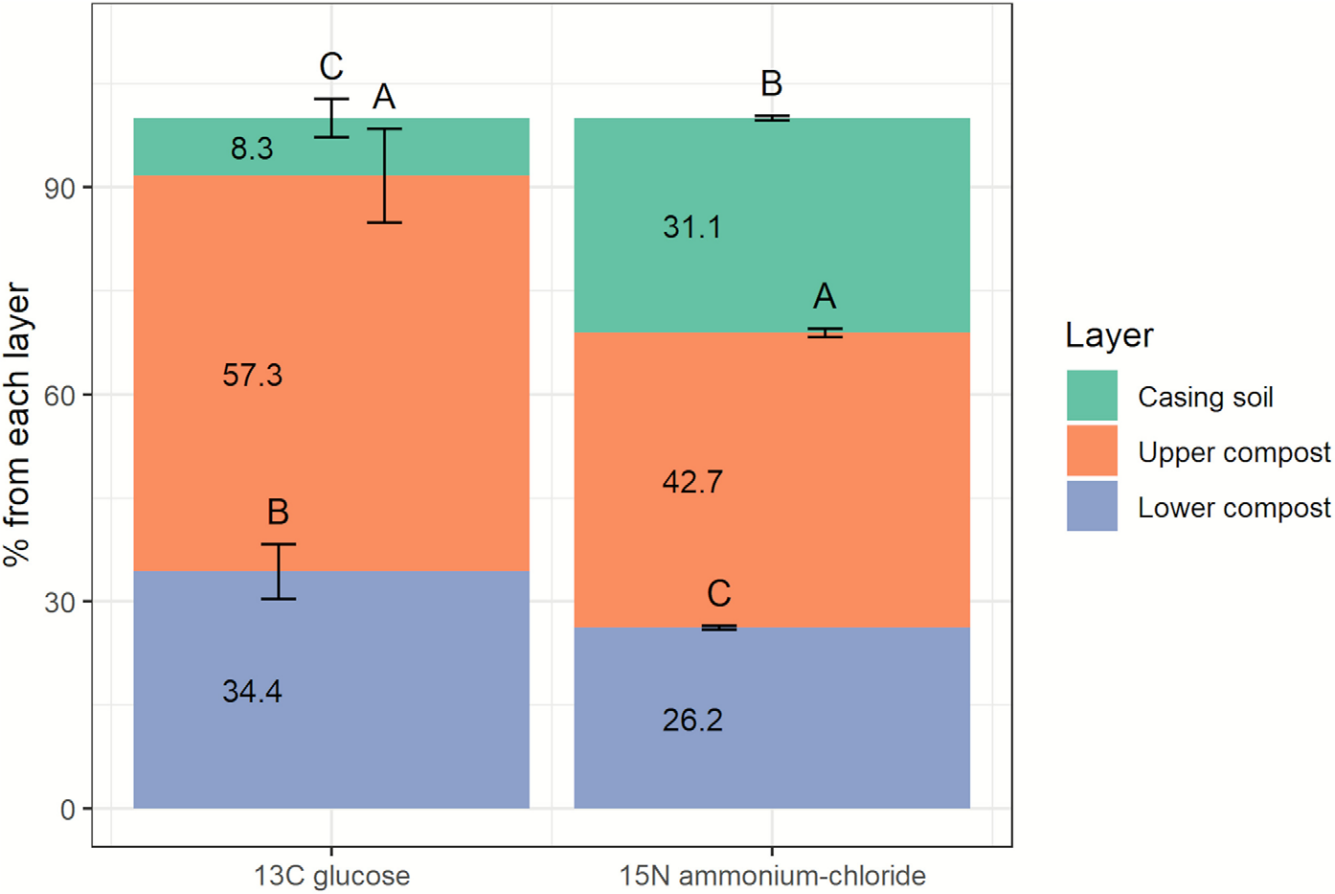
Mean relative tracer uptake by forming pins from each substrate layer. Glucose‐derived ^13^C and ammonium‐derived ^15^N measured in the pins of the incubation carried out in small square plates (ES4, Figure 1). On the *X*‐axis are the sources of tracer while the *Y*‐axis shows the percentage of total tracer uptake by the pins from each layer: Green for casing soil, orange for top compost layer and blue for bottom compost layer. This was calculated according to Equation S1 concomitant with the indicated assumptions, based on the excess tracer atom fractions within the mushrooms. One‐way ANOVA with Tukey post hoc test was performed, comparing tracer enrichment in mushrooms resulting from labelling a specific layer, for ^13^C‐glucose and ^15^N‐ammonium chloride independently.

**FIGURE 10:**
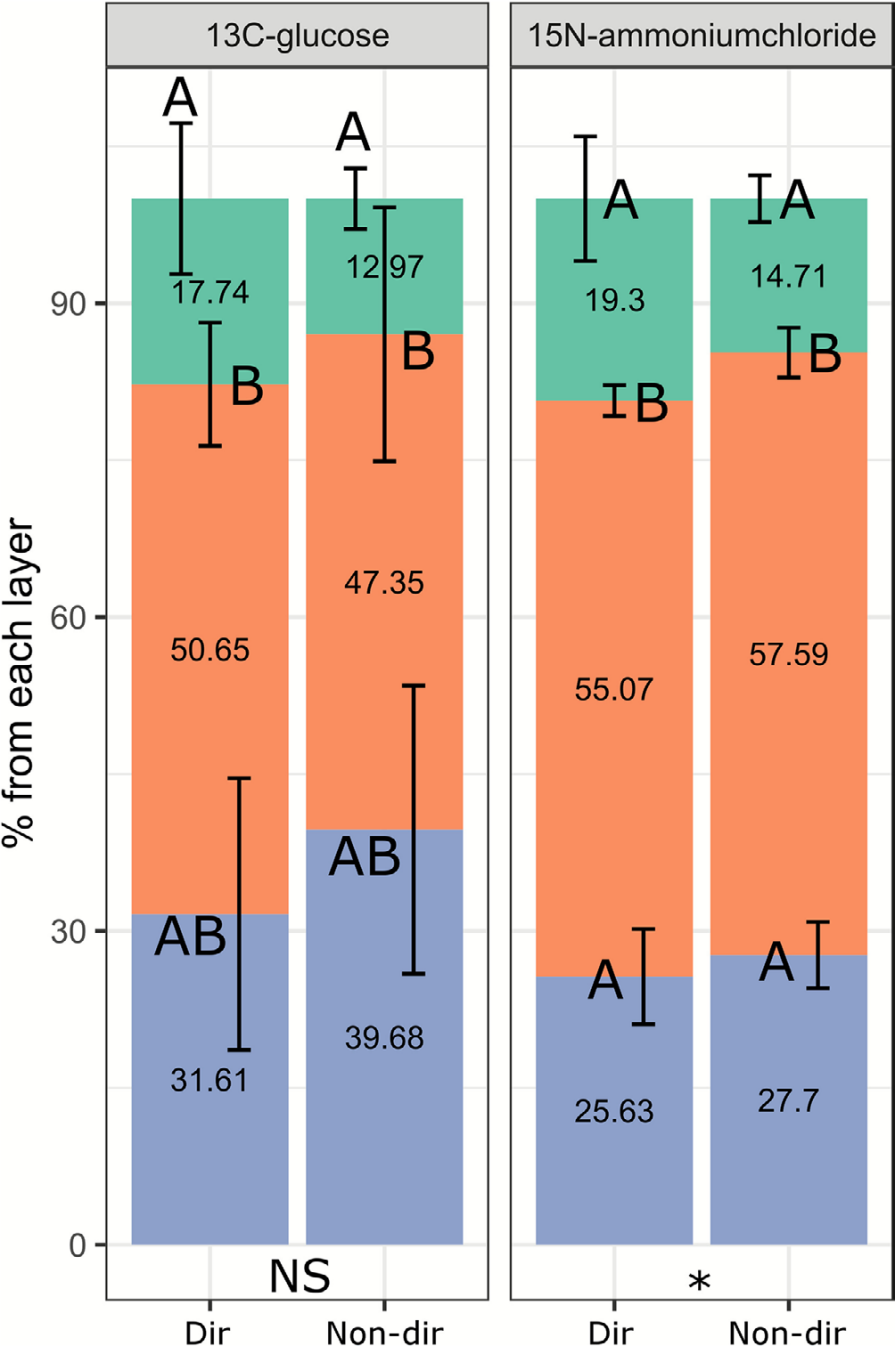
Mean relative tracer uptake by developing mushrooms from each substrate layer (ES5, Figure 1). *X*‐axis shows the mycelial network architecture: (Dir) directional with many cords, (Non‐dir) non‐directional with few cords. *Y*‐axis shows the percentage of total tracer uptake by the mushrooms from each layer: Green for casing soil, orange for top layer and blue for bottom layer. This was calculated according to Equation S1 concomitant with the indicated assumptions, based on the excess tracer atom fractions within the mushrooms. Two‐way ANOVA with Tukey post hoc tests was performed, with Dir/Non‐dir (indicated with asterisk or NS for significant and non‐significant effects, respectively) and layers (indicated with letters) as factors, comparing tracer enrichment in mushrooms resulting from labelling of a specific layer, for ^13^C‐glucose and ^15^N‐ammonium chloride independently.

We investigated from which compost layers first flush fruiting bodies can take carbon and nitrogen tracers for growth. To this end, either the casing, top, or bottom compost layer were labelled with ^15^N and ^13^C tracers. ^15^N and ^13^C enrichment of the growing mushroom pins showed that the top compost layer mainly contributed to the transport of glucose‐derived ^13^C to these forming fruiting body initials, while the casing was the least utilised (Figure 9) (ES4, Figure 1). For ^15^N enrichment, the top compost layer played the biggest role in delivering tracer‐derived nitrogen to the growing mushrooms, followed by the casing layer and then the bottom compost layer. Next, we studied the feeding of fully grown mushrooms in upscaled incubations (ES5, Figure 1). These samples showed that the top compost layer is the most significant source of tracer‐derived ^13^C and ^15^N for the first flush of mushrooms, followed by the lower layer of compost and lastly the layer of casing soil (Figure 10). It is important to note that these findings reflect the uptake and translocation of specific tracers (glucose‐derived ^13^C and ammonium‐derived ^15^N) rather than a generalised nutrient source, as the enrichment observed in mushrooms is influenced by the mycelium's efficiency in uptake, transport, and assimilation of the administered tracers. Additionally, higher uptake percentages were observed in the casing layer when the network developed directionally (Figure 10, Dir). However, the network architecture in the compost (many or few cords; directional versus non‐directional, respectively) did not impact the transfer of the added tracer to the mushrooms substantially. Moreover, relatively more ammonium‐derived nitrogen was taken from the top compost layer than glucose‐derived carbon. This indicates that the mycelial network feeding the mushrooms differentially scavenges nitrogen and carbon tracers.

### 3.6 Cords Possess Distinct Cell Types That Facilitate Nutrient Storage and Transport

**FIGURE 11:**
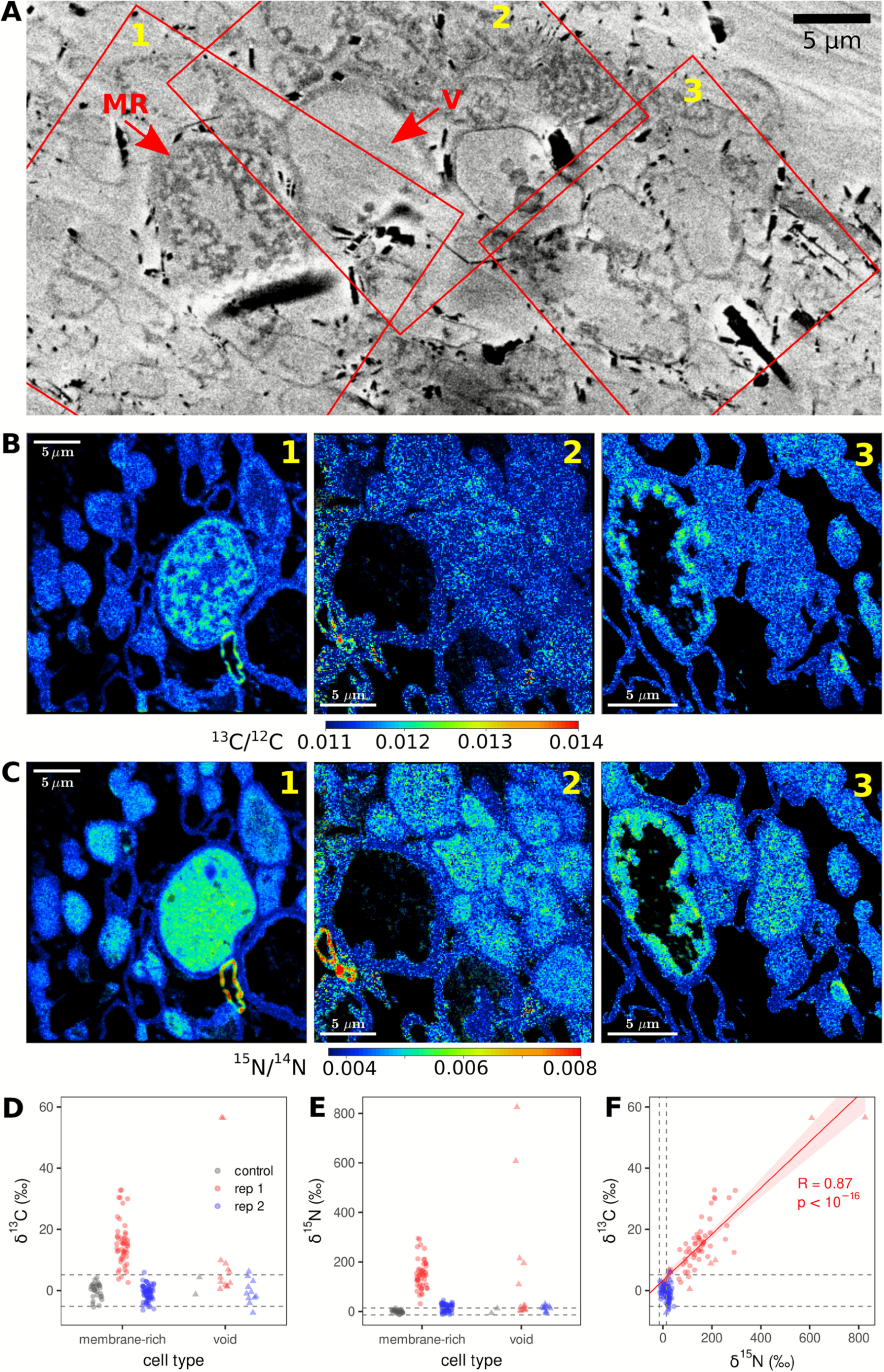
SEM and NanoSIMS imaging of fungal cords to analyse nutrient transport. Two colonies were allowed to grow from rye spawns and connect to each other via cords. One rye spawn inoculum was labelled with ^13^C and ^15^N labelled substrates and incubated to allow tracer transport through the cords. (A) SEM image of the cord cross section. Shown are different morphologies of the hyphal cells (MR = hyphae rich in inner membranes, V = void hyphae). (B, C) Images of the ^13^C/^12^C and ^15^N/^14^N isotope ratios obtained by NanoSIMS. Images show localization of the tracer enrichment in the cytosol or inner membranes but generally not in the cell wall. Only image 1 and 2 each show one hypha with a cell wall enriched in ^13^C and ^15^N. Images 1–3 correspond to areas marked by red squares in panel A, the corresponding elemental maps (CN, S, P and O) are shown in Figure S6. (D, E). Isotope enrichment in hyphal cells. Data points show cell‐specific δ^13^C and δ^15^N values in individual cells from two replicate fungal cords (rep 1 and 2), separately for two of the identified cell morphotypes (void vs. internal membrane‐rich). The enrichments are expressed relative to the control cells, which were grown in natural media without added tracer. Dashed horizontal lines indicate boundaries corresponding to +/− 2× SD of the δ^13^C and δ^15^N values in the control cells. Thus, data points above the upper boundary line correspond to cells with a significant isotope enrichment. (F) Correlation analysis between the δ^13^C and δ^15^N values in individual hyphal cells. Significant correlation (*R* = 0.87, *p* < 10^−16^, Pearson) is found for the isotopically enriched cells from replicate cord 1 (red line), but not in replicate cord 2 (not shown).

Cords serve as highways for water and nutrient transport to zones of growth such as the growing colony periphery and the developing mushrooms (Herman et al. 2020). Here we made an attempt to reveal which cell types within cords facilitate resource transport employing a combined SEM‐nanoSIMS imaging (ES3, Figure 1). Cross sections of cords unveiled a diverse array of cell types and sizes intricately woven together to form hyphal bundles, that is, cords (Figure 11A, Figure S4). These cells exhibited varying widths, ranging from 3 to 15 μm, and presented distinct characteristics such as (i) small and (ii) large cells devoid of cytoplasm, (iii) large cells with small internal membrane structures in which tracer‐derived carbon and nitrogen accumulated, (iv) small cells with lower levels of homogeneous tracer‐derived C and N distribution and (v) cells with organelles containing inclusion bodies, rich in phosphorous and oxygen or a combination of nitrogen, carbon and sulphur, and rarely a combination of all five while the cytosol contained a homogeneous distribution of P, N, C and S (Figure 11, Figures S5 and S6). Moreover, certain cell types with large vacuoles with inclusion bodies were observed to cluster together, not exceeding a diameter of 7 μm, and exhibited a higher relative phosphorus content (Figure S5). These observations suggest that the hyphae constituting a cord specialise and could have distinct functions.

For cells from cord replicate 1, the δ^13^C and δ^15^N values were significantly greater than the corresponding values in the control cells (Figure 11D,E, rep 1 vs. control). Additionally, the δ^15^N and δ^13^C values were significantly correlated (Figure 11F) and, on average, the δ^15^N values surpassed the δ^13^C values. By and large, void cells had lower δ^15^N and δ^13^C values than membrane‐rich cells. Some cells did not show any enrichment. On the one hand the resin could have replaced the existing tracers (in case of carbon); on the other hand, these cells might have a different function than the membrane‐rich cells and actually possess lower ^15^N and ^13^C enrichment. We observed another cell type that harboured large vacuoles containing P and O, or C, N and S rich granules (Figure S5). This suggests a possible division of tasks within cords, where some hyphae are involved in nutrient (nitrogen and carbon) transport (inner membrane‐rich cells, Figure 11A, ‘MR’), others presumably in water transport (void cells, Figure 11A, ‘V’), and still others in storing phosphate (P and O) and other nutrients (C, N and S) within vacuoles (vacuole rich cells, Figure S5). The presence of two cells (Figure 11B,C; images 1 and 2) with cell walls enriched in ^13^C and ^15^N indicates that cords still grow new hyphal branches after their initial formation. In contrast, ‘MR’ cells (Figure 11) showed lower enrichment in their cell walls compared to their cytoplasm. This indicates that these cells had been produced before they took up tracer. Cells in cord replicate 2 did not show significant isotope enrichment above the natural levels, suggesting that not all cords equally contribute to nutrient transport. Overall, cords possess different cell types that likely have distinct functions. The large vessel hyphae containing internal membranes predominantly mediate carbon and nitrogen transport.

## 4 Discussion

### 4.1 Mechanisms of Nutrient Translocation in the Mycelium

In the present study we investigated translocation patterns of tracer‐derived nitrogen and carbon in axenic cultures and the compost bed of *A. bisporus*. In general, fungi are adapted to break down complex organic materials through the secretion of extracellular enzymes, enabling the absorption of simpler molecules. For this reason, simple sources of nitrogen and carbon (^15^N‐ammonium chloride and ^13^C‐glucose) were chosen as the carriers of the isotope tracers introduced in the system (Leigh et al. 2008). We focussed on the impact of growth, network architecture and water uptake on nutrient translocation.

After uptake of the tracer carriers, isotope tracers are likely quickly incorporated into biomolecules, which will alter their transport dynamics by processes such as sequestration, respiration, or secretion. In the axenic conditions we could clearly attribute tracer transport to the *A. bisporus* mycelium, however in compost cultures this was more challenging. For instance, the compost microbiome may have taken up and sequestered the tracers, thereby (temporarily) lowering the accessibility to *A. bisporus* mycelium. On the other hand, *A. bisporus* is known to lyse and feed on bacteria (Fermor and Wood 1981), which likely results in the observed decline in gram negative bacteria during the spawn run (PIII) (Vos et al. 2017). Additionally, bacteria are known to use fungal mycelium as ‘travelling highways’ (Keshari and Kanwar 2021), and nematodes are found moving through the compost (Vita 2025; Keshari and Kanwar 2021) and feed on fungi/bacteria. These processes complicate tracer cycling and may obscure/influence the contribution of *A. bisporus* to tracer uptake and possibly their translocation. Nevertheless, by carefully selecting and controlling experimental conditions, we could still attribute tracer transport to *A. bisporus* mycelium in compost, as discussed below.

In axenic conditions, three control conditions were employed to evaluate the contribution of alternative nutrient translocation mechanisms than cytoplasmic mass‐flow (Figure S1). In the first control condition, where uncovered MEA ring plates were used, ^13^C showed no translocation, while ^15^N exhibited translocation. The possibility that ammonium could convert to ammonia, degas and subsequently dissolve somewhere else, could explain the observed pattern. Ammonium preferentially converts to ammonia in a basic environment and MEA is acidic (pH 5.4) (Emerson et al. 1975). However, this conversion occurs gradually, so part of the present ammonium could still undergo the conversion, degassing and dissolution cycle. The second control condition comprised a PC membrane covering the medium and revealed minor translocation of ^13^C, limited to the adjacent rings in both Ring 1 and Ring 5 labelling conditions, suggesting a restricted diffusion of carbon via the membrane. However, enrichment of ^15^N was observed in all rings, indicating potentially stronger nitrogen diffusion effects. This could be explained by their 10‐fold difference in molecular weight, hence ~60‐fold difference in diffusion coefficient in water (glucose: approximately 6.7E‐10 m^2^/s at 25°C (Kreft et al. 2013); ammonium: approximately 1.135E‐9 m^2^/s at 25°C (Ribeiro et al. 2001)), and by the ionic nature of ammonium. Possibly these membranes are covered with a water film explaining the observed diffusion. The third control condition involving a membrane and a heat‐killed *A. bisporus* colony demonstrated enrichment of both ^13^C and ^15^N in all rings. Notably, when Ring 1 was labelled, more translocation occurred away from the ring compared to when labelling Ring 5, suggesting a preference for passive translocation from the centre to the periphery via the mycelium. Thus, growth is not the sole prerequisite for tip‐directed translocation in this experimental setup. One hypothesis is that the observed translocation happens via external wicking via the hyphae, but why would this have a directional preference? In agreement with this, mycelia have been shown to transport water from humid to arid areas across their network (Worrich et al. 2017; Guhr et al. 2015), but it is unclear what factor drove wicking in our experiments. It is important to note that when colonies are alive, they respire and metabolise the added labelled substrates, so less is available for translocation. Thus, the increased translocation in dead colonies can be the result of higher tracer availability.

The above results (Figure S1) and results with living mycelium (Figures 2 and 3) indicate that most of the ^13^C translocation happened externally and not via growth‐induced intercellular mass flow. Moreover, the majority of the initial ^13^C could not be detected after 7 days. Therefore, we anticipate that most ^13^C is quickly respired and/or stored in living biomass, so that it is not available for transport. Indeed, when comparing Figures 2 and 3 about 80%, 50% and 25% of carbon was respired by the mycelium of Ring 1, 3 and 5, respectively, if we ignore the relatively small contribution of tracer translocation. The translocation of ^15^N from the colony centre to periphery was higher when living mycelium was present (Figures 2 and 3) than in the control conditions (Figure S1), therefore indicating growth‐induced intercellular mass flow. However, the majority of ^15^N translocation happened through passive processes (Figure 4), such as potentially trough wicking effects or intra‐ or extracellular diffusion via the mycelium. Similar results were previously obtained with *Phanerochaete velutina* using an amino acid analog (Tlalka, Bebber, et al. 2008). However, this is in contrast to (Heaton et al. 2020) where modelling suggested that growth was the main component explaining the induction of mass flow. Since *A. bisporus* grows very slowly on MEA (it takes about 1 month to fully colonise a standard Petri dish), the translocation observed may therefore more depend on passive processes. Fungal cell walls can bind micro‐nutrients (Kleijburg et al. 2023). If this is a transient process, it is tempting to speculate that the translocation we observed may happen extracellularly across the cell wall lining the hyphae.

Water uptake was found to be necessary for the translocation of the non‐metabolizable 2‐deoxy‐D‐glucose (Figure 5), suggesting that this may also be the case for stable isotope tracers. The fact that non‐metabolizable carbon (2‐deoxy‐D‐glucose) did extensively translocate and metabolizable ^13^C‐glucose did not much translocate further indicates that respiration and metabolism limited carbon transport.

### 4.2 Translocation in Colonies Grown in Compost

In spawn‐inoculated compost, when the tracer was applied to the middle or the rim of the Petri dish, both ^13^C and ^15^N exhibited similar translocation in both directions (Figure 6). While the translocation from centre towards periphery was expected as this was the direction of growth of the mycelium (Herman et al. 2020), the observed substantial translocation from the periphery towards the centre was not expected. This phenomenon could be attributed to extended incubation times (14 days), which likely resulted in complete cessation of hyphal growth, and overtaken by diffusion/wicking as the main translocation mechanism. Alternatively, mycelium first colonises fast and sparse, and when the substrate is fully colonised, a second wave of branching happens especially in the colony centre, which could induce a reversed flow. Supporting the former, we did not find translocation of ^14^C‐aminoisobutyric acid (AIB) in compost during shorter incubation times of 5.5 days (Herman et al. 2020). Moreover, it is known that bacteria can use fungal hyphae as highways to travel long distances (Simon et al. 2015). If this event occurs in the compost as well, part of the tracer could have been distributed by the migration of bacteria from one area of the dish to another. It is also possible that the translocation of tracer happens faster than the growth rate (Herman et al. 2020). Moreover, in addition to bacteria and *A. bisporus*, an active fungal population is also present in the compost (Vita 2025). This population could also affect tracer translocation patterns during long incubations (Carrasco et al. 2020). While the deuterated water distributed mostly randomly in the compost bed (Figure S2A), this was not seen for the C and N translocation patterns (Figures 7 and 8). Therefore, most likely, water was translocated extracellularly through evaporation and condensation rather than wicking along the surface of the straw particles. This hypothesis is supported by the fact that 60% of the compost volume is composed of humid air (Jurak 2015) and it has been shown that water does condense within the compost (Vos et al. 2021).

### 4.3 Mass Flow During Colonisation and Fructification in the Compost Bed

In the compost bed, the translocation of tracer‐derived carbon and nitrogen towards the upper layers was found to be dependent on growth, occurring only during the colonisation of the casing layer or the formation of fruiting bodies (Figures 7 and 8). Less tracer translocation was observed in ES5 (Figure 8) than in ES4 (Figure 7). However, it is important to note that the tracer incubation period was shorter in ES5 (3 days) than in ES4 (30 days; Figure 1), which could explain the different observations.

There was almost no translocation of tracers from the casing to the compost (Figure 7). In contrast, tracers did translocate within the compost and from the compost to the casing. It is possible that fungal cords, which are more abundant in the casing soil, do not take up, transport and subsequently release nutrients in the compost environment. The tracers detected in the compost and casing are likely present in the mycelium. When analysing the pins (primordia), the top compost layer mostly contributed to feeding the pins with tracers. For ^15^N, casing contributed much more than for ^13^C in feeding the forming pins (Figure 9). However, the difference in size and molecular weight of the two tracers could have favoured the diffusion of ammonia over glucose in the moisture‐rich environment and cord‐rich fungal network of the casing. The mushrooms showed consistent results between glucose‐derived ^13^C and ammonium‐derived ^15^N (Figure 10). In both cases the top compost layer was the main source of tracers feeding the mushrooms, followed by the bottom layer, and casing contributed the least. These results are in line with the Sonnenberg et al. study that showed that the upper layer of the compost was mostly utilised for mushroom formation up to the first flush (Sonnenberg et al. 2022). However, the natural C and N present in the substrate could be in different forms than that used to introduce the tracers, and their utilisation may depend on the network colonisation density, substrate degradation efficiency, and/or competition with its microbiome, etc.

### 4.4 The Impact of the Network Architecture

We employed two types of fungal growth in this study: directional growth and non‐directional growth, with the former resulting in increased cord formation (Figure 1, ES5). In the absence of an active sink for nutrients, such as growing mushrooms, there is no need for nutrient transport throughout the mycelial network. This was consistently observed in both round and square dish cultures where *A. bisporus* was not present (Figures 6C,D and 7C,D). Discrepancies in enrichment, particularly in the labelled layers, could be partially attributed to degassing of ammonium. The compost microbiome is known to convert ammonium into ammonia (Fermor et al. 1985). When the layers were labelled, the mycelium and/or substrate did not immediately take up the stock solution (Figure 1, ES5); it took several hours for the drops to be completely absorbed due to the hydrophobicity of the cultures. Additionally, it is unclear to what extent the mycelium absorbs the nutrients, as it is challenging to differentiate between the substrate and the mycelium within the substrate. Tracer uptake by mushrooms also differed between directional‐ and non‐directional growth. Less uptake from the lower compost layer was observed in directional growth than non‐directional growth for glucose‐derived ^13^C (Figure 10). Cords, which are more abundant in directional growth, might take up less nutrients due to their low surface to volume ratio. Since the tracer was added above the PIII‐compost inoculum (Figure 1, ES5), where mostly cords were present, it is not surprising that less nutrients were taken up (Figure 10). In contrast, in a similar study by Herman et al. (2020) radioactive ^14^C‐AIB tracer was added on the PIII‐end compost inoculum having fine mycelium, instead of on the cords as in our study. As a result, 5× more tracer was found in the mushrooms when directional growth was employed compared to non‐directional growth. This suggests that fine mycelium is well equipped to take up nutrients, while cords do not take up well, but are better equipped to bulk transport nutrients. This is substantiated by the fact that casing, which almost exclusively contains cords, fed mushrooms least with tracer over a 3 days time window (Figure 10). PIII‐end compost was already 16 days old when the cultures were inoculated, while in directional growth, the age was zero at filling. In directional growth, after 2–3 weeks of colonisation, the top compost layer is much younger than the bottom, as the hyphae have been present in the bottom layer for more time. Older compost may have less nutrient availability since more nutrients could be taken up for longer. However, the old compost could also have been more degraded and thus more nutrients could have been freed. Surprisingly, although the casing is poorer in nutrients than the compost, it still had a percentage of 13%–20% contributing to feeding the fruiting bodies, when labelled with easily accessible nutrients (glucose/ammonium). Normally casing does not contribute to feeding the mushrooms (unpublished results), since it is nutrient poor. Although liquid fertiliser could be envisioned as a supplementation strategy of casing this would likely disrupt the osmotic balance between the casing and the mushrooms, and would result in infections or inhibit the pinning process.

### 4.5 Specialised Hyphae in Cords Mediate Nutrient Storage and Bulk Nutrient Transport

Cords are the main long‐distance transport mycelial structure responsible for delivering nutrients to the growing mushrooms (Herman et al. 2020). Moreover, the fruiting bodies originate from enlarged cords (Baars et al. 2020). Imaging of hyphae in cross sections of cords revealed higher levels of ^13^C and ^15^N tracers in hyphae having high amounts of internal membrane structures (Figure 11). This is in line with a hypothesis that transport relies on tubular vacuoles and vesicle movements (Darrah et al. 2006). Moreover, some cells that appeared to have a thick cell wall and void lumen proved to be not or on average much lower enriched for ^13^C and ^15^N, and did not contain C, N, S, P or O suggesting they do not transport nutrients. In addition, we found hyphae containing organelles with inclusions rich in phosphorus and oxygen, sulphur, carbon and nitrogen (Figure S6). These organelles are likely vacuoles, since vacuoles are known to store nutrients within granules in the form of polyphosphate (White and Brown 1979; Yang et al. 2017). In plants, proteins are stored in specialised vacuoles containing protein bodies (Ibl et al. 2014). The sulphur, C and N granules we observed could be protein bodies (Figure S5).

In general, ^15^N atom fractions were higher than those of ^13^C. This could be the result of a lower enrichment for carbon in respect to nitrogen. There was more ^15^N enrichment (*δ* values of up to 800‰) than ^13^C enrichment (*δ* values of up to 60‰). It could be that the carbon‐rich resin substituted part of the ^13^C‐tracer. Therefore, higher amounts of ^13^C tracers are advisable when imaging samples embedded in resin. Additionally, ^13^C could have been respired as seen in the axenic conditions (Figure 3B).

In summary, a differentiation in morphology and function could be observed in the hyphae of the cords. This differentiation resembles a plant‐like vascular system with empty wide, potentially dead cells in cords (Figure 11) transporting water (xylem) (Pratt et al. 2021) and the ones rich in internal membranes transporting nutrients (phloem) (Lough and Lucas 2006). Moreover, some hyphae are likely utilised for nutrient storage as seen in the cells with polyphosphate and organic nitrogen, sulphur and carbon rich inclusions in their large vacuoles (Figure S5). These might serve as companion cells to maintain the cord functioning in analogy with xylem parenchyma cells (Chaffey et al. 2003). Moreover, they could possibly load nutrients into hyphae that facilitate bulk transport when there is a large sink, such as during mushroom formation. It remains unclear how specific hyphae within cords are loaded and unloaded with nutrients and whether water transport is mediated by specific hyphae (e.g., void cells) or the same hyphae that transport nutrients (e.g., internal membrane‐rich cells). More work is required to specifically pinpoint the exact functions of these specific cell types that we identified here.

## Author Contributions

Conceptualization: M.M.V., J.B.M.M., R.B., N.N.v.d.W. Investigation: M.M.V., T.L.G.v.V., N.N.v.d.W., D.D.E. Formal analysis: M.M.V., T.L.G.v.V., D.D.E., L.P., R.B. Supervision: J.B.M.M., L.P., R.B. Funding acquisition: J.B.M.M., R.B., L.P. Writing – original draft: M.M.V., T.L.G.v.V., D.D.E., L.P., J.B.M.M., R.B.

## Funding

This work was financed by the NWO TTW grant ‘Traffic control’, grant number 15493, by the NWO TTW Vidi grant ‘Feed me’, grant number 18920 and the Netherlands Earth System Science Centre. The funding bodies had no involvement in study design; data collection, management, analysis and interpretation; or the decision to submit for publication.

## Conflicts of Interest

The authors declare no conflicts of interest.

## Supporting information


**Figure S1:** Enrichment of medium for each control condition. *X*‐axis shows the sampled ring, *Y*‐axis shows tracer found as a percentage of total tracer added to the plate. ^15^N enrichment in control condition (A) 1, (B) 2, (C) 3. ^13^C enrichment in control condition (D) 1, (E) 2, (F) 3. The three control conditions are: (1) nothing on top to test for external diffusion within the medium and translocation via degassing and dissolution, (2) PC membrane on top to test for external diffusion via the membrane, and (3) dead mycelium on top to test for internal diffusion and wicking along the hyphae. The titles of each graph indicate the labelled ring 1 or 5. Note the high range of values in panel A for ring 3 when ring 1 was labelled. This is due to one of the two replicates being abnormally high, possibly due to spilling of the label during labelling. One‐way ANOVA with Tukey post hoc test was performed, comparing tracer enrichment between rings when one ring was labelled.

**Figure S2:** Variability in deuterium enrichments in compost and fungal PLFAs. (A) shows the enrichment in deuterium of the bulk PIII‐end compost, while (B) represents the enrichment of the fungal PLFAs. Error bars are SD (*n* = 3). (A) deuterated water was added to the bottom layer of the compost, the labels on the *X*‐axis indicate the sampled areas, FB fruiting bodies, CAS casing, TOP top layer of the compost, MID middle layer of the compost, BOT bottom layer of the compost (Experimental design ES5, see Figure 1). (B) A and B are replicates of the same condition where the same amount of deuterated water was added to the centre (Experimental design ES2, see Figure 1). (A) One‐way ANOVA with Tukey post hoc test was performed, but showed no significant differences.

**Figure S3:** Translocation of ^15^N between different substrate layers in (A) directional and (B) non‐directional cultures. In directional cultures, a thin layer of phase III‐end compost was used as inoculum abutted to a large layer of PII‐end compost, whereas in non‐directional cultures, spawn was homogeneously mixed with phase II‐end compost to inoculate the substrate. The headers indicate which layer was labelled with ^15^N‐ammonium chloride. The *X*‐axis indicates which layer was sampled (1: casing soil, 2: upper compost, 3: lower compost), and the *Y*‐axis shows the percentage ^15^N detected relative to the total tracer added. Bars are colour‐coded to indicate whether mushrooms were allowed to grow (fructification, green) or not (no fructification, orange). Error bars represent standard deviation (SD) based on three replicates (*n* = 3). Two‐way ANOVA with Tukey post hoc test was performed, with layers (indicated by letters) and fructification/no fructification as factors, comparing tracer enrichment between layers when one layer was labelled. No significant effect of fructification was detected.

**Figure S4:** *A. bisporus* cord morphology and composition. (A) SEM imaging of cords formed on polycarbonate membranes which were placed on MEA. (B) Cords formed in 1:4 wet weight mixture of casing and PII‐end compost. (C) A cross section of a cord. (D) Cross sections of two cords within the same resin section with cords area and hyphal area variation and distribution in red for the top section and purple for the bottom one, the purple section was chosen for further analysis with nanoSIMS (Figure 11, rep 2) (E) Cross sections of a cord with cord area and hyphal area variation and distribution, this section was chosen for further analysis with nanoSIMS (Figure 11, rep 1; Figures S5 and 6). These hyphae ranged from rich inclusions (□) or void (◊) (D) to void (∆) or internal membrane rich (*) (E).

**Figure S5:** Elemental maps of different cell types within the fungal cords. In contrast to images shown in Figure S6, this figure was obtained for cells that did not show isotope enrichment (from cord replicate 2). Field of view with void (∆) and internal membrane‐rich cells (*). Cell with vacuoles containing phosphate inclusions (○, blue colour) and C, N, S and O inclusions (orange colour). Both sections contain hyphal cells with a dense cytoplasm, containing all measured elements, but less oxygen (◊). Scale bars correspond to 5 μm.

**Figure S6:** Elemental maps of hyphal cells within cords obtained by nanoSIMS. Shown are RGB overlays as well as images of individual secondary ions ^12^C^14^N, ^32^S, ^31^P and ^16^O (log‐transformed) for the same fields of view (marked 1–3) as the isotope ratio images shown in Figure 11, that is, in cells that showed significant isotope enrichment (from cord replicate 1). Note that the grey‐scale of the ^16^O ion count images was adjusted such that the low‐intensity features are visible. Due to this adjustment, ^16^O ion counts in hotspots corresponding to oxalic acid precipitates appear oversaturated. Scale bars correspond to 5 μm.

**Table S1:** Ring volumes of the ring plate (see also Levin et al. (2007).

**Table S2:** Atom fractions (%) for deuterium oxide labelled rings (ES1, Figure 1) in three control conditions (i–iii) (see Material and Methods). Top row indicates which ring was labelled (R1 = Ring 1 and R5 = Ring 5). Left column indicates which rings were sampled.

## Data Availability

The data that support the findings of this study are openly available in Figshare at https://figshare.com/s/75d1753c97d2660f4bb2.
