# Supplementary figures and images for: Mechanisms of Differential Resource Uptake and Translocation in Agaricus bisporus
Source: Environ Microbiol. 2026 Jan 8;28(1):e70222. doi: 10.1111/1462-2920.70222 (PMC12783971; doi:10.1111/1462-2920.70222)

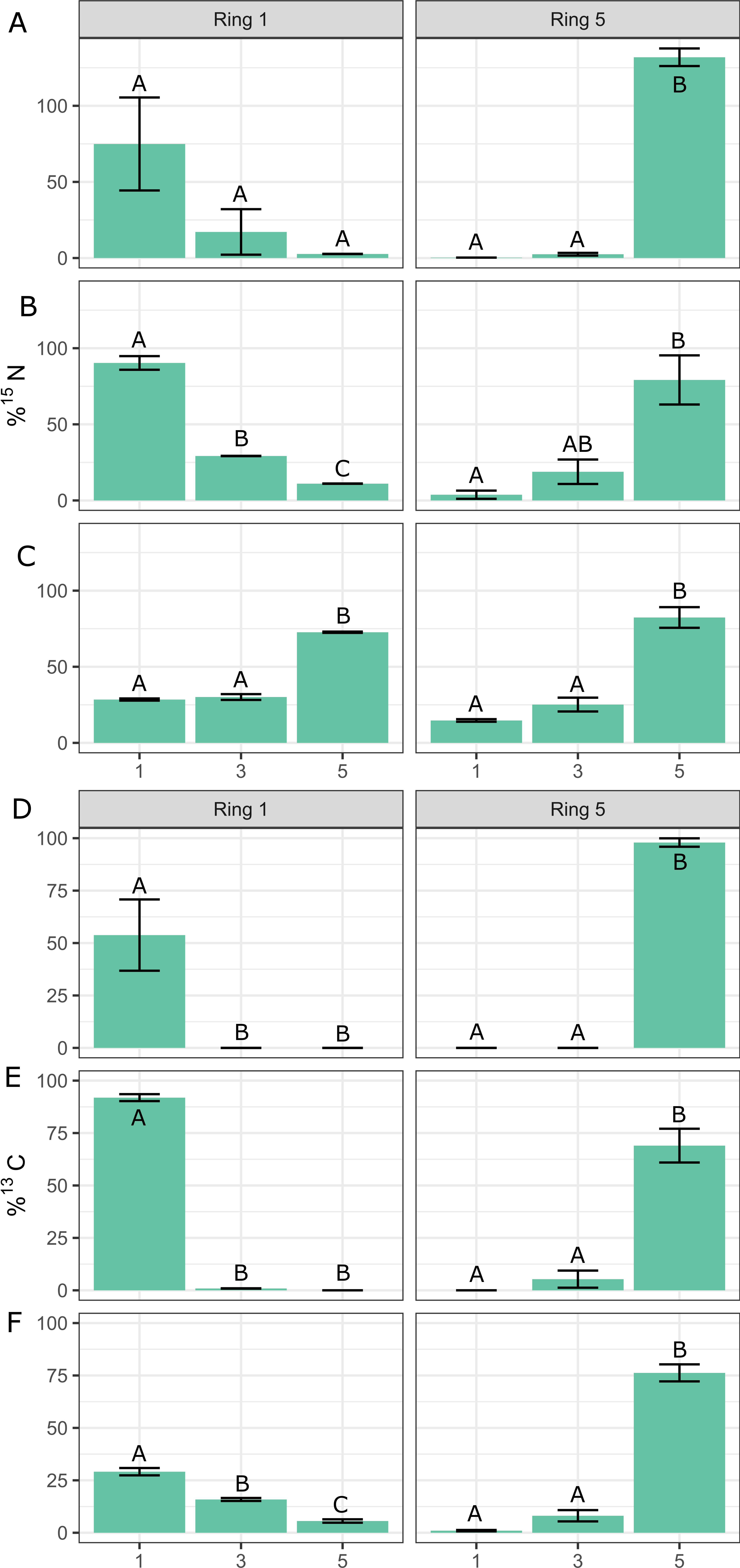

Supplement: Supplementary file 1 — Figure S1: Enrichment of medium for each control condition. X‐axis shows the sampled ring, Y‐axis shows tracer found as a percentage of total tracer added to the plate. 15N enrichment in control condition (A) 1, (B) 2, (C) 3. 13C enrichment in control condition (D) 1, (E) 2, (F) 3. The three control conditions are: (1) nothing on top to test for external diffusion within the medium and translocation via degassing and dissolution, (2) PC membrane on top to test for external diffusion via the membrane, and (3) dead mycelium on top to test for internal diffusion and wicking along the hyphae. The titles of each graph indicate the labelled ring 1 or 5. Note the high range of values in panel A for ring 3 when ring 1 was labelled. This is due to one of the two replicates being abnormally high, possibly due to spilling of the label during labelling. One‐way ANOVA with Tukey post hoc test was performed, comparing tracer enrichment between rings when one ring was labelled. [file EMI-28-e70222-s008.png]

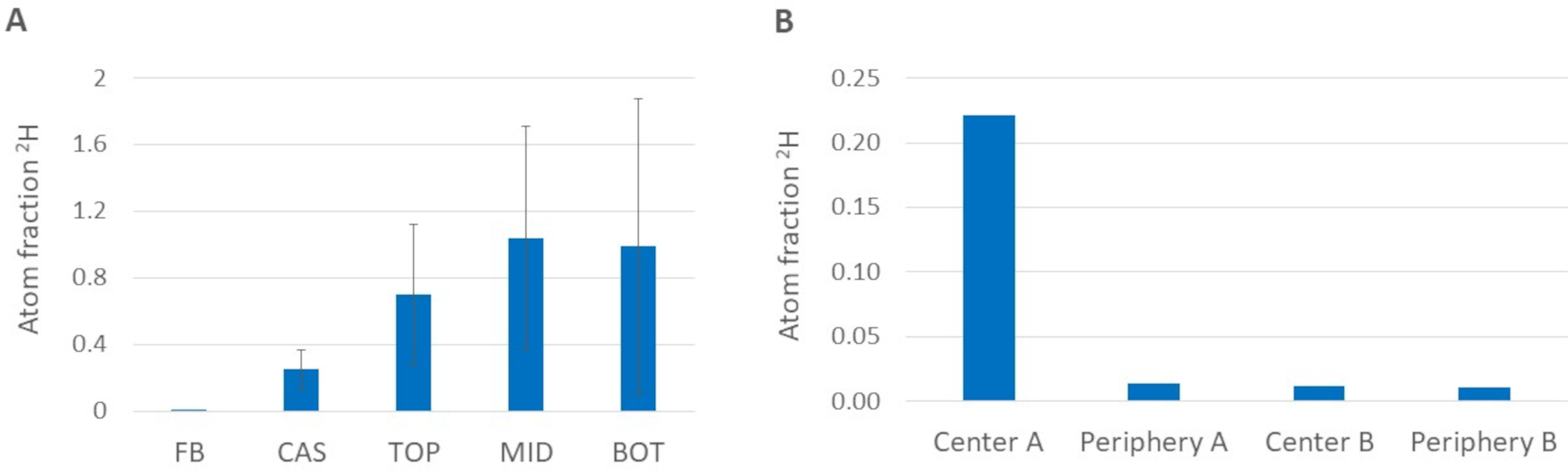

Supplement: Supplementary file 2 — Figure S2: Variability in deuterium enrichments in compost and fungal PLFAs. (A) shows the enrichment in deuterium of the bulk PIII‐end compost, while (B) represents the enrichment of the fungal PLFAs. Error bars are SD (n = 3). (A) deuterated water was added to the bottom layer of the compost, the labels on the X‐axis indicate the sampled areas, FB fruiting bodies, CAS casing, TOP top layer of the compost, MID middle layer of the compost, BOT bottom layer of the compost (Experimental design ES5, see Figure 1). (B) A and B are replicates of the same condition where the same amount of deuterated water was added to the centre (Experimental design ES2, see Figure 1). (A) One‐way ANOVA with Tukey post hoc test was performed, but showed no significant differences. [file EMI-28-e70222-s003.png]

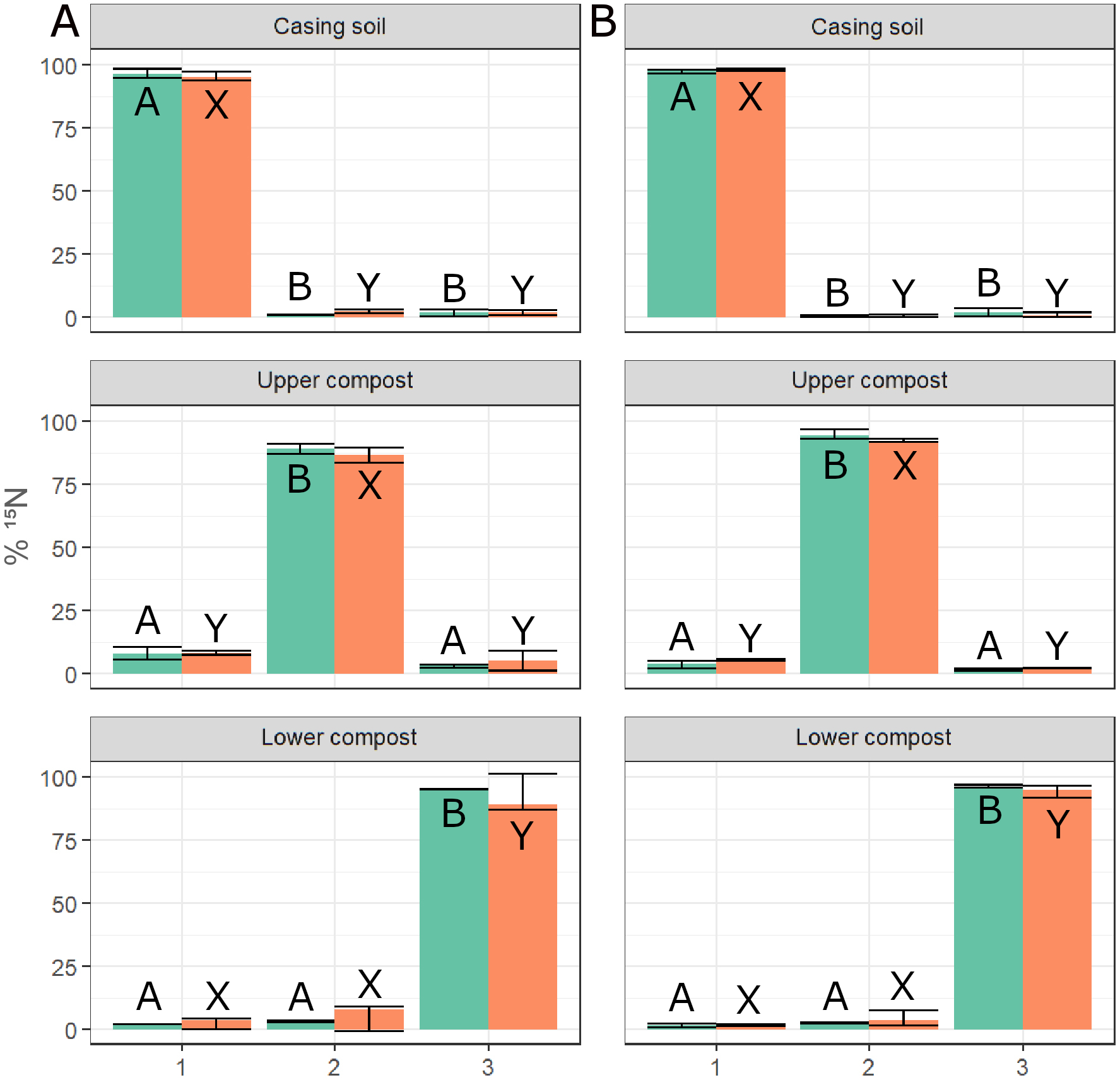

Supplement: Supplementary file 3 — Figure S3: Translocation of 15N between different substrate layers in (A) directional and (B) non‐directional cultures. In directional cultures, a thin layer of phase III‐end compost was used as inoculum abutted to a large layer of PII‐end compost, whereas in non‐directional cultures, spawn was homogeneously mixed with phase II‐end compost to inoculate the substrate. The headers indicate which layer was labelled with 15N‐ammonium chloride. The X‐axis indicates which layer was sampled (1: casing soil, 2: upper compost, 3: lower compost), and the Y‐axis shows the percentage 15N detected relative to the total tracer added. Bars are colour‐coded to indicate whether mushrooms were allowed to grow (fructification, green) or not (no fructification, orange). Error bars represent standard deviation (SD) based on three replicates (n = 3). Two‐way ANOVA with Tukey post hoc test was performed, with layers (indicated by letters) and fructification/no fructification as factors, comparing tracer enrichment between layers when one layer was labelled. No significant effect of fructification was detected. [file EMI-28-e70222-s006.png]

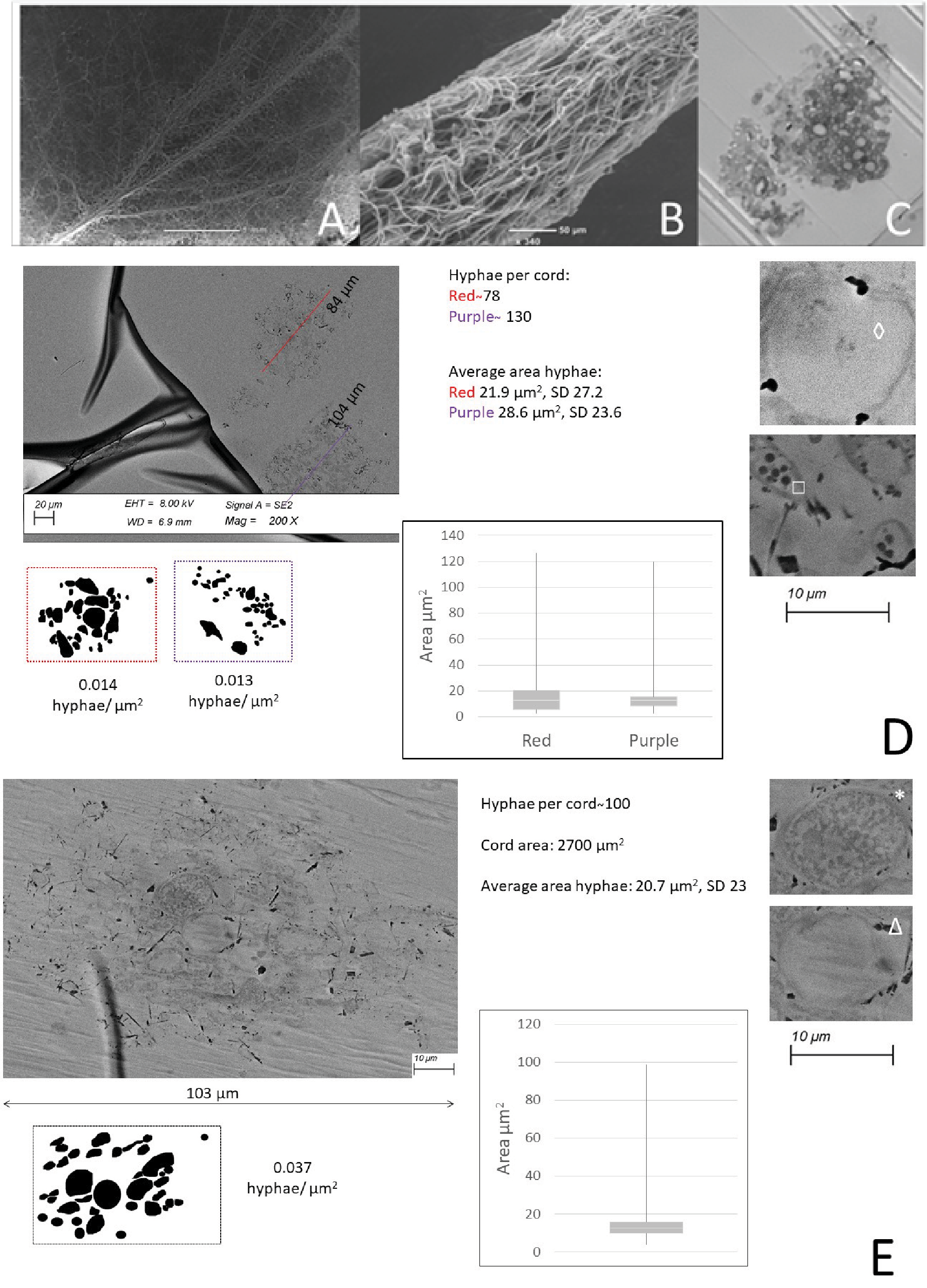

Supplement: Supplementary file 4 — Figure S4: A. bisporus cord morphology and composition. (A) SEM imaging of cords formed on polycarbonate membranes which were placed on MEA. (B) Cords formed in 1:4 wet weight mixture of casing and PII‐end compost. (C) A cross section of a cord. (D) Cross sections of two cords within the same resin section with cords area and hyphal area variation and distribution in red for the top section and purple for the bottom one, the purple section was chosen for further analysis with nanoSIMS (Figure 11, rep 2) (E) Cross sections of a cord with cord area and hyphal area variation and distribution, this section was chosen for further analysis with nanoSIMS (Figure 11, rep 1; Figures S5 and 6). These hyphae ranged from rich inclusions (□) or void (◊) (D) to void (∆) or internal membrane rich (*) (E). [file EMI-28-e70222-s005.png]

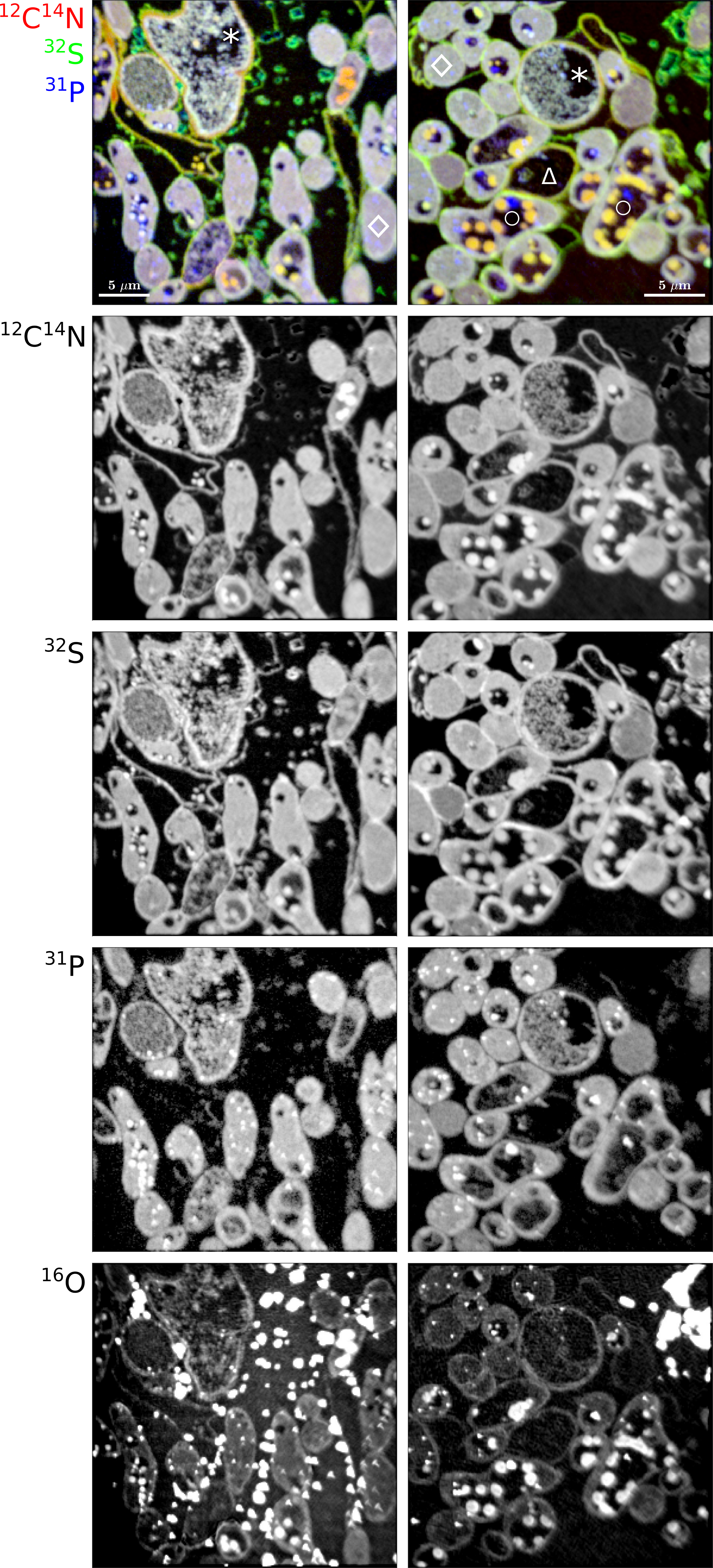

Supplement: Supplementary file 5 — Figure S5: Elemental maps of different cell types within the fungal cords. In contrast to images shown in Figure S6, this figure was obtained for cells that did not show isotope enrichment (from cord replicate 2). Field of view with void (∆) and internal membrane‐rich cells (*). Cell with vacuoles containing phosphate inclusions (○, blue colour) and C, N, S and O inclusions (orange colour). Both sections contain hyphal cells with a dense cytoplasm, containing all measured elements, but less oxygen (◊). Scale bars correspond to 5 μm. [file EMI-28-e70222-s001.png]

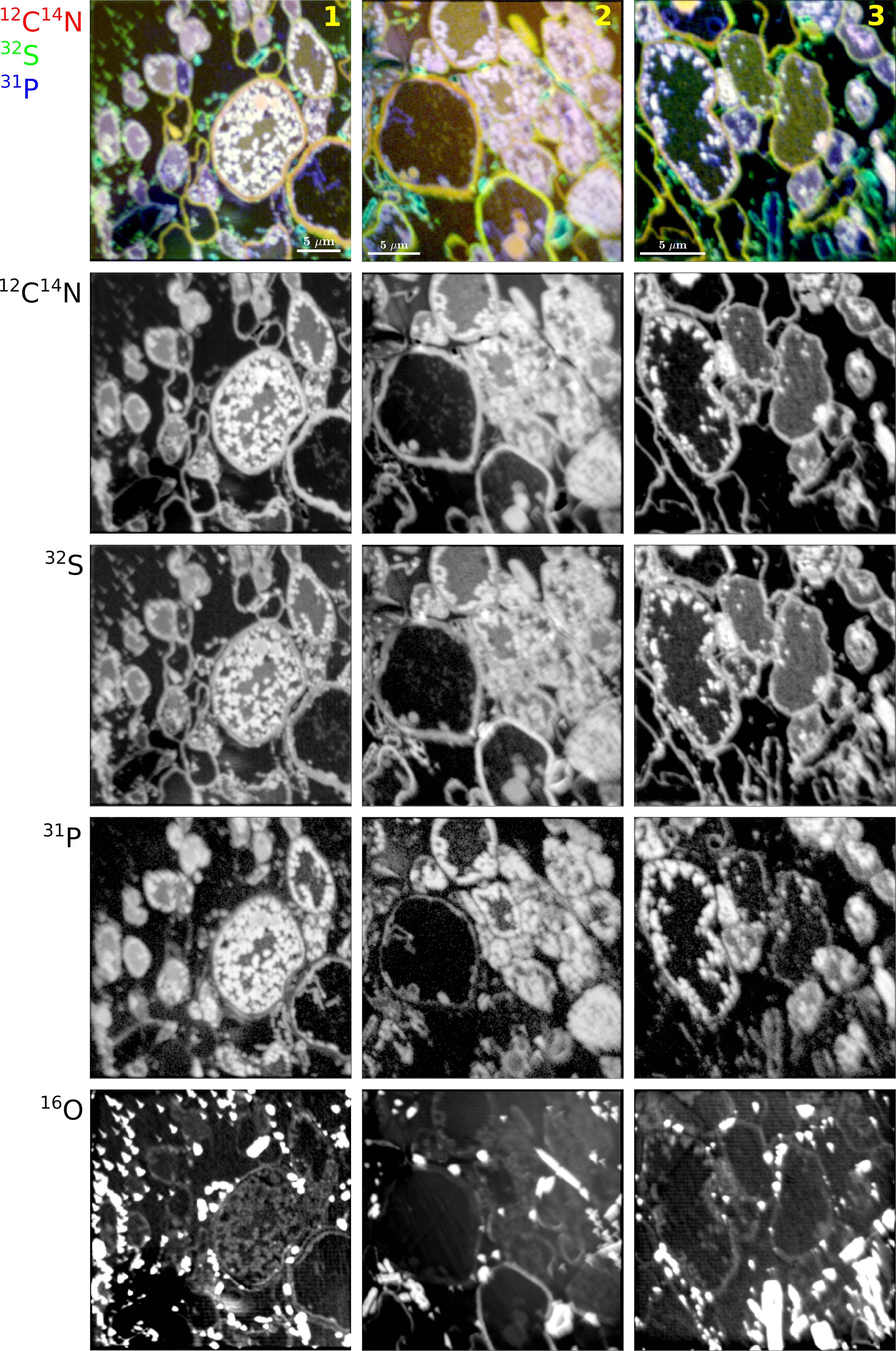

Supplement: Supplementary file 6 — Figure S6: Elemental maps of hyphal cells within cords obtained by nanoSIMS. Shown are RGB overlays as well as images of individual secondary ions 12C14N, 32S, 31P and 16O (log‐transformed) for the same fields of view (marked 1–3) as the isotope ratio images shown in Figure 11, that is, in cells that showed significant isotope enrichment (from cord replicate 1). Note that the grey‐scale of the 16O ion count images was adjusted such that the low‐intensity features are visible. Due to this adjustment, 16O ion counts in hotspots corresponding to oxalic acid precipitates appear oversaturated. Scale bars correspond to 5 μm. [file EMI-28-e70222-s007.png]
