# Supplementary material for: Mechanisms of Differential Resource Uptake and Translocation in Agaricus bisporus
Source: Environ Microbiol. 2026 Jan 8;28(1):e70222. doi: 10.1111/1462-2920.70222 (PMC12783971; doi:10.1111/1462-2920.70222)
Supplement: Supplementary file 7 — Table S1: Ring volumes of the ring plate (see also Levin et al. (2007). [file EMI-28-e70222-s002.docx]

***Table S1****: Ring volumes of the ring plate (see also Levin et al., (2007)* (44)*)*

| **Ring number** | **Ring volume (ml)** |
| --- | --- |
| 1 | 0.83 |
| 2 | 1.47 |
| 3 | 2.21 |
| 4 | 2.91 |
| 5 | 3.47 |
