# Supplementary material for: Mechanisms of Differential Resource Uptake and Translocation in Agaricus bisporus
Source: Environ Microbiol. 2026 Jan 8;28(1):e70222. doi: 10.1111/1462-2920.70222 (PMC12783971; doi:10.1111/1462-2920.70222)
Supplement: Supplementary file 8 — Table S2: Atom fractions (%) for deuterium oxide labelled rings (ES1, Figure 1) in three control conditions (i–iii) (see Material and Methods). Top row indicates which ring was labelled (R1 = Ring 1 and R5 = Ring 5). Left column indicates which rings were sampled. [file EMI-28-e70222-s004.docx]

***Table S2****: Atom fractions (%) for deuterium oxide labelled rings (ES1, Fig. 1) in three control conditions (i-iii) (see Material and Methods). Top row indicates which ring was labelled (R1 = ring 1 and R5 = ring 5). Left column indicates which rings were sampled.*
